# Supplementary material for: Extreme QTL mapping enables the identification of novel zinc toxicity response loci in Drosophila melanogaster
Source: Genetics. 2025 Aug 21;231(3):iyaf173. doi: 10.1093/genetics/iyaf173 (PMC12606420; doi:10.1093/genetics/iyaf173)
Supplement: iyaf173_Supplementary_Data [file iyaf173_supplementary_data.zip › Supplementary_Material_GENETICS-2025-308275.pdf]

Supplementary Table 1: Number of female flies emerging from each bottle. (Note that all control bottles received 24µl of pipetted eggs except for those bottles of Replicate 8 that received 48µl. And all zinc bottles received 48µl of eggs except for those bottles of Replicate 8 that received 60µl).

Replicate	Treatment	Volume Eggs (ul)	Bottle	Num. Adult Females
R1	Con	24	1	124
R1	Con	24	2	143
R1	Con	24	3	136
R1	Con	24	4	120
R2	Con	24	1	90
R2	Con	24	2	124
R2	Con	24	3	145
R2	Con	24	4	139
R3	Con	24	1	93
R3	Con	24	2	96
R3	Con	24	3	98
R3	Con	24	4	114
R4	Con	24	1	118
R4	Con	24	2	120
R4	Con	24	3	104
R4	Con	24	4	137
R5	Con	24	1	131
R5	Con	24	2	95
R5	Con	24	3	101
R5	Con	24	4	110
R6	Con	24	1	132
R6	Con	24	2	136
R6	Con	24	3	97
R6	Con	24	4	121
R7	Con	24	1	159
R7	Con	24	2	160
R7	Con	24	3	146
R7	Con	24	4	135
R8	Con	48	1	251
R8	Con	48	2	249
R8	Con	48	3	253
R8	Con	48	4	264
R9	Con	24	1	180
R9	Con	24	2	190
R9	Con	24	3	177
R9	Con	24	4	207
R10	Con	24	1	154
R10	Con	24	2	190
R10	Con	24	3	155
R10	Con	24	4	226
R11	Con	24	1	151
R11	Con	24	2	143
R11	Con	24	3	156
R11	Con	24	4	188
R12	Con	24	1	171
R12	Con	24	2	168
R12	Con	24	3	222
R12	Con	24	4	178

Replicate	Treatment	Volume Eggs (ul)	Bottle	Num. Adult Females
R1	Zinc	48	1	33
R1	Zinc	48	2	31
R1	Zinc	48	3	21
R1	Zinc	48	4	13
R1	Zinc	48	5	11
R1	Zinc	48	6	16
R1	Zinc	48	7	23
R1	Zinc	48	8	7
R1	Zinc	48	9	38
R1	Zinc	48	10	20
R1	Zinc	48	11	32
R1	Zinc	48	12	24
R1	Zinc	48	13	13
R1	Zinc	48	14	16
R1	Zinc	48	15	16
R1	Zinc	48	16	12
R1	Zinc	48	17	17
R1	Zinc	48	18	7
R1	Zinc	48	19	8
R1	Zinc	48	20	18
R2	Zinc	48	1	25
R2	Zinc	48	2	15
R2	Zinc	48	3	15
R2	Zinc	48	4	14
R2	Zinc	48	5	21
R2	Zinc	48	6	19
R2	Zinc	48	7	33
R2	Zinc	48	8	14
R2	Zinc	48	9	31
R2	Zinc	48	10	15
R2	Zinc	48	11	16
R2	Zinc	48	12	15
R2	Zinc	48	13	31
R2	Zinc	48	14	15
R2	Zinc	48	15	11
R2	Zinc	48	16	23
R2	Zinc	48	17	16
R3	Zinc	48	1	11
R3	Zinc	48	2	8
R3	Zinc	48	3	9
R3	Zinc	48	4	15
R3	Zinc	48	5	19
R3	Zinc	48	6	15
R3	Zinc	48	7	12
R3	Zinc	48	8	36
R3	Zinc	48	9	19
R3	Zinc	48	10	18
R3	Zinc	48	11	11
R3	Zinc	48	12	20
R3	Zinc	48	13	24
R3	Zinc	48	14	12
R3	Zinc	48	15	19
R3	Zinc	48	16	24
R3	Zinc	48	17	4
R3	Zinc	48	18	23

Replicate	Treatment	Volume Eggs (ul)	Bottle	Num. Adult Females
R4	Zinc	48	1	12
R4	Zinc	48	2	6
R4	Zinc	48	3	12
R4	Zinc	48	4	8
R4	Zinc	48	5	9
R4	Zinc	48	6	15
R4	Zinc	48	7	14
R4	Zinc	48	8	12
R4	Zinc	48	9	11
R4	Zinc	48	10	9
R4	Zinc	48	11	8
R4	Zinc	48	12	15
R4	Zinc	48	13	13
R4	Zinc	48	14	10
R4	Zinc	48	15	12
R4	Zinc	48	16	15
R4	Zinc	48	17	12
R4	Zinc	48	18	21
R4	Zinc	48	19	12
R4	Zinc	48	20	23
R4	Zinc	48	21	11
R5	Zinc	48	1	9
R5	Zinc	48	2	6
R5	Zinc	48	3	11
R5	Zinc	48	4	17
R5	Zinc	48	5	12
R5	Zinc	48	6	27
R5	Zinc	48	7	20
R5	Zinc	48	8	16
R5	Zinc	48	9	25
R5	Zinc	48	10	21
R5	Zinc	48	11	15
R5	Zinc	48	12	13
R6	Zinc	48	1	5
R6	Zinc	48	2	21
R6	Zinc	48	3	16
R6	Zinc	48	4	4
R6	Zinc	48	5	23
R6	Zinc	48	6	7
R6	Zinc	48	7	11
R6	Zinc	48	8	5
R6	Zinc	48	9	26
R6	Zinc	48	10	18
R6	Zinc	48	11	23
R6	Zinc	48	12	0
R6	Zinc	48	13	6
R6	Zinc	48	14	2
R6	Zinc	48	15	3
R6	Zinc	48	16	3
R6	Zinc	48	17	3
R6	Zinc	48	18	3
R6	Zinc	48	19	8
R6	Zinc	48	20	3

Replicate	Treatment	Volume Eggs (ul)	Bottle	Num. Adult Females
R7	Zinc	48	1	28
R7	Zinc	48	2	27
R7	Zinc	48	3	18
R7	Zinc	48	4	25
R7	Zinc	48	5	20
R7	Zinc	48	6	21
R7	Zinc	48	7	24
R7	Zinc	48	8	15
R7	Zinc	48	9	23
R7	Zinc	48	10	15
R7	Zinc	48	11	19
R7	Zinc	48	12	29
R7	Zinc	48	13	32
R7	Zinc	48	14	22
R7	Zinc	48	15	29
R7	Zinc	48	16	42
R7	Zinc	48	17	14
R7	Zinc	48	18	20
R7	Zinc	48	19	23
R7	Zinc	48	20	30
R8	Zinc	60	1	35
R8	Zinc	60	2	34
R8	Zinc	60	3	23
R8	Zinc	60	4	37
R8	Zinc	60	5	25
R8	Zinc	60	6	33
R8	Zinc	60	7	25
R8	Zinc	60	8	26
R8	Zinc	60	9	29
R8	Zinc	60	10	26
R8	Zinc	60	11	33
R8	Zinc	60	12	25
R9	Zinc	48	1	16
R9	Zinc	48	2	21
R9	Zinc	48	3	16
R9	Zinc	48	4	11
R9	Zinc	48	5	30
R9	Zinc	48	6	34
R9	Zinc	48	7	24
R9	Zinc	48	8	17
R10	Zinc	48	1	25
R10	Zinc	48	2	27
R10	Zinc	48	3	23
R10	Zinc	48	4	24
R10	Zinc	48	5	14
R10	Zinc	48	6	23
R10	Zinc	48	7	20
R10	Zinc	48	8	36
R10	Zinc	48	9	19
R10	Zinc	48	10	28
R10	Zinc	48	11	25
R10	Zinc	48	12	26

Replicate	Treatment	Volume Eggs (ul)	Bottle	Num. Adult Females
R11	Zinc	48	1	26
R11	Zinc	48	2	33
R11	Zinc	48	3	12
R11	Zinc	48	4	21
R11	Zinc	48	5	29
R11	Zinc	48	6	40
R11	Zinc	48	7	26
R11	Zinc	48	8	29
R11	Zinc	48	9	15
R11	Zinc	48	10	49
R11	Zinc	48	11	12
R11	Zinc	48	12	19
R12	Zinc	48	1	13
R12	Zinc	48	2	49
R12	Zinc	48	3	43
R12	Zinc	48	4	21
R12	Zinc	48	5	28
R12	Zinc	48	6	32
R12	Zinc	48	7	23
R12	Zinc	48	8	30
R12	Zinc	48	9	27
R12	Zinc	48	10	29
R12	Zinc	48	11	36
R12	Zinc	48	12	20
R12	Zinc	48	13	12
R12	Zinc	48	14	16

Supplementary Table 2: Details of all 12 X-QTL replicates.

Replicate	Non-Selection Control Treatment				Zinc Selection Treatment				Estimated Number of Females Exposed to Zinc Treatment **	Estimated Fraction of Females Exposed to Zinc and Survived (%) **
	Number of Bottles	Embryos/ Bottle (µl)	Total Emerged Females per Replicate	Emerged Females per µl	Number of Bottles	Embryos/ Bottle (µl)	Total Emerged Females per Replicate *	Emerged Females per µl		
R1	4	24	523	5.448	20	48	376	0.392	5230	7.189
R2	4	24	498	5.188	17	48	329	0.403	4233	7.772
R3	4	24	401	4.177	18	48	299	0.346	3609	8.285
R4	4	24	479	4.990	21	48	260	0.258	5029.5	5.169
R5	4	24	437	4.552	12	48	192	0.333	2622	7.323
R6	4	24	486	5.063	20	48	190	0.198	4860	3.909
R7	4	24	600	6.250	20	48	476	0.496	6000	7.933
R8	4	48	1017	5.297	12	60	351	0.488	3813.75	9.204
R9	4	24	754	7.854	8	48	169	0.440	3016	5.603
R10	4	24	725	7.552	12	48	290	0.503	4350	6.667
R11	4	24	638	6.646	12	48	311	0.540	3828	8.124
R12	4	24	739	7.698	14	48	379	0.564	5173	7.327

* This is also the number of females present in each pool from which we obtained DNA for sequencing libraries.

** To enhance efficiency, we did not manually count the number of embryos tested per bottle/replicate/treatment, and instead pipetted aliquots of embryos resuspended in 1X PBS into each bottle. Given we do not know the number of embryos per bottle, we are estimating the zinc-selection pressure exerted on females based on the emergence of control females within each replicate. For each treatment the “Emerged Females per µl” values are the “Total Number of Emerged Females per Replicate” divided by the total volume of embryos (in µl) used per replicate (“Number Bottles” multiplied by “Embryos/Bottle (µl)”). The “Estimated Number of Females Exposed to Zinc Treatment” is the total volume of embryos (in µl) used for the Zinc Selection Treatment multiplied by the number of “Emerged Females per µl” for the Non-Selection Control Treatment. And the “Estimated Fraction of Females Exposed to Zinc and Survived (%)” is calculated as the ratio of the “Emerged Females per µl” for the two treatments (Zinc Selection divided by Non-Selection Control).

Supplementary Table 3: Number of female flies used to isolate DNA per treatment/replicate, and the average genome sequencing coverage obtained per pool.

**Treatment**	**Replicate**	**Num. Adult Females**	**Coverage (X)**
Con	1	376	70.2
Con	2	329	79.1
Con	3	299	73.5
Con	4	260	59.8
Con	5A	192	72.6
Con	5B	192	62.9
Con	6A	190	59.5
Con	6B	190	60.5
Con	7	476	65.1
Con	8A	351	54.9
Con	8B	351	57.4
Con	9	169	57.8
Con	10	290	63.3
Con	11A	311	80.4
Con	11B	311	60.0
Con	12A	379	59.2
Con	12B	379	68.9
Zinc	1	376	58.6
Zinc	2	329	63.2
Zinc	3	299	64.2
Zinc	4	260	74.7
Zinc	5	192	86.4
Zinc	6	190	51.7
Zinc	7	476	82.0
Zinc	8	351	72.7
Zinc	9	169	80.2
Zinc	10	290	69.1
Zinc	11	311	83.8
Zinc	12	379	63.5

Supplementary Text 1: DNA isolation protocol.

Requires Qiagen Puregene Cell Kit (Cat. No. 158046) for the Cell Lysis Solution, RNase A, and Protein Precipitation Solution, and Qiagen Buffer EB (Cat. No. 19086).

1. Add 5 glass beads and 1ml of 1X PBS to a 2ml screwtop tube containing the pool of flies. Then homogenize the flies by placing tube in a MiniBeadBeater-96, running the instrument for two 45 second intervals.

2. Pour homogenate into a 15ml tube, then rinse out the original screwtop tube with 1ml of cold Cell Lysis Solution (CLS) and then pour into the 15ml tube. Repeat as necessary.

3. Add 7 ml of CLS, minus the amount used to rinse the screwtop tube (see Step 2), to the 15 ml tube. Invert to mix, and then incubate at 65°C in a hybridization oven for 30 minutes.

4. Cool to room temperature and then move 600 µl of homogenate to 1.7ml tube. (The remainder of the homogenate can be discarded, or frozen at −20°C in case additional DNA is needed subsequently).

5. Add 3 µl of undiluted RNase A solution to the 600 µl of homogenate, mix, and then incubate in a hot block at 37°C for 40 minutes. Rapidly cool sample to room temperature by placing on ice for 1 minute.

6. Add 200 µl protein precipitation solution to the sample, vortex on high speed for 20 seconds, and place sample on ice for 5 minutes. Centrifuge sample at ~20,000 g for 3 minutes.

7. Move 600 µl of the supernatant to a new 1.7 ml tube containing 600 µl of Isopropanol and mix by inverting. Centrifuge at ~20,000 g for 1 minute, and gently pour off supernatant.

8. Add 600 µl of 70% ethanol, and invert tube to wash pellet. Centrifuge at ~20,000 g, for 1 minute, and gently pour off supernatant. Invert tube on absorbent paper and air dry for 15 minutes.

9. Add 50 µl EB buffer and incubate at 65 °C for 1 hour. Flick the tube to resuspend DNA.

Supplementary Table 4: Candidate genes in X-QTL intervals.

X-QTL	Gene Symbol	Gene Name	FlyBase ID
A	ben	bendless	FBgn0000173
B	Gart **	GART trifunctional enzyme	FBgn0000053
B	Mnn1	Menin 1	FBgn0031885
B	CG4496	-	FBgn0031894
B	Ndae1	Na^+-driven anion exchanger 1	FBgn0259111
C	Trpm	Transient receptor potential cation channel, subfamily M	FBgn0265194
C	Vha36-1 **	Vacuolar H+ ATPase 36kD subunit 1	FBgn0022097
C	Vha14-1 **	Vaculoar H+ ATPase 14kD subunit 1	FBgn0262512
D	GluRIB	Glutamate receptor IB	FBgn0264000
D	Hsp22	Heat shock protein 22	FBgn0001223
D	MTF-1	Metal response element-binding Transcription Factor-1	FBgn0040305
E	CG14302 **	-	FBgn0038647
E	Mekk1	Mekk1	FBgn0024329
E	Octα2R	α2-adrenergic-like-octopamine receptor	FBgn0038653
E	Xrp1	Xrp1	FBgn0261113
E *	ATPsynD	ATP synthase, subunit D	FBgn0016120
F	RanBP3	Ran binding protein 3	FBgn0039110
F	Nup98-96	Nucleoporin 98-96kD	FBgn0039120
F	Ndc1	Nuclear division cycle 1	FBgn0039125
G	Fer1HCH ***	Ferritin 1 heavy chain homologue	FBgn0015222
G	Fer2LCH ***	Ferritin 2 light chain homologue	FBgn0015221
G	dj-1β	dj-1β	FBgn0039802
G *	pHCl-2	pH-sensitive chloride channel 2	FBgn0039840
G *	CG11318	-	FBgn0039818

Information sourced from FB2022_06 (Gramates et al. 2022).

* These 3 genes are each just outside the formal 3-LOD support interval for our final set of X-QTL (see Table 1). These genes are included in our candidate set because they were within X-QTL intervals using an earlier version of our X-QTL analysis framework, and remain very close to the final set of X-QTL intervals we report.

** No TRiP UAS-RNAi strains were available for these target genes.

*** Knockdown of these genes via *mex1*-GAL4-driven, midgut-specific RNAi led to almost zero adult emergence in the water control treatment in initial tests, suggesting gene knockdown is lethal. These genes were not assayed further.

Supplementary Text 2. Ingredients and brief protocol for Macdonald lab cornmeal-yeast-molasses fly media.

28.5-liters water
318-g agar (Genesee Scientific; 66-111. This is based on a gel strength of 960 g/cm2, and will change depending on the batch)

- Add water to steam kettle, turn on electric mixer, and slowly add agar
- Bring mix to a boil

3,200-ml molasses (Genesee Scientific; 62-117)

- Reduce the kettle pressure to reduce the heat slightly
- Add molasses, and bring mix back to a boil

4-liters water
1,460-g inactive dry yeast (Genesee Scientific; 62-107)

- Mix in bucket using paint-stirring drill attachment

4-liters water
2,600-g yellow cornmeal (Genesee Scientific; 62-101)

- Mix in bucket using paint-stirring drill attachment
- Add both the water/yeast and water/cornmeal mixes to the steam kettle
- Bring mix back to boil, and simmer for ~15-min
- Release pressure from steam kettle, but continue to stir with electric mixer

330-ml water
259-ml propionic acid (ThermoFisher; A258-500)
31-ml phosphoric acid (85%; ThermoFisher; A242-500)

- Pour mix into kettle

400-ml 95% ethanol
40-g tegosept (Genesee Scientific; 20-258)

- Dissolve tegosept in ethanol
- Pour mix into kettle
- Fill vials/bottles

Supplementary Figure 1: Comparing duplicate control pools reveals few differences.

For five X-QTL replicates – R5, R6, R8, R11 and R12 – we generated sequencing libraries from two independent pools of unselected, control females ("A" and "B" pools for each replicate). We tested for differentiation in the haplotype frequencies between the pairs of control pools following the same analysis used to compare zinc-selected and unselected populations, treating the "A" and "B" sets of pools as different treatments. No peaks reached above a $-\log_{10}(P)$ threshold of 4. On the X chromosome there is one aberrant window with a $-\log_{10}(P)$ value >5, but this point does not fall within the interval implicated by QTL-A (the only X-linked zinc resistance locus we identified). Equally, the peak on chromosome 3L that has a $-\log_{10}(P)$ value of ~3.5 does not full within the LOD drop interval for QTL-D.

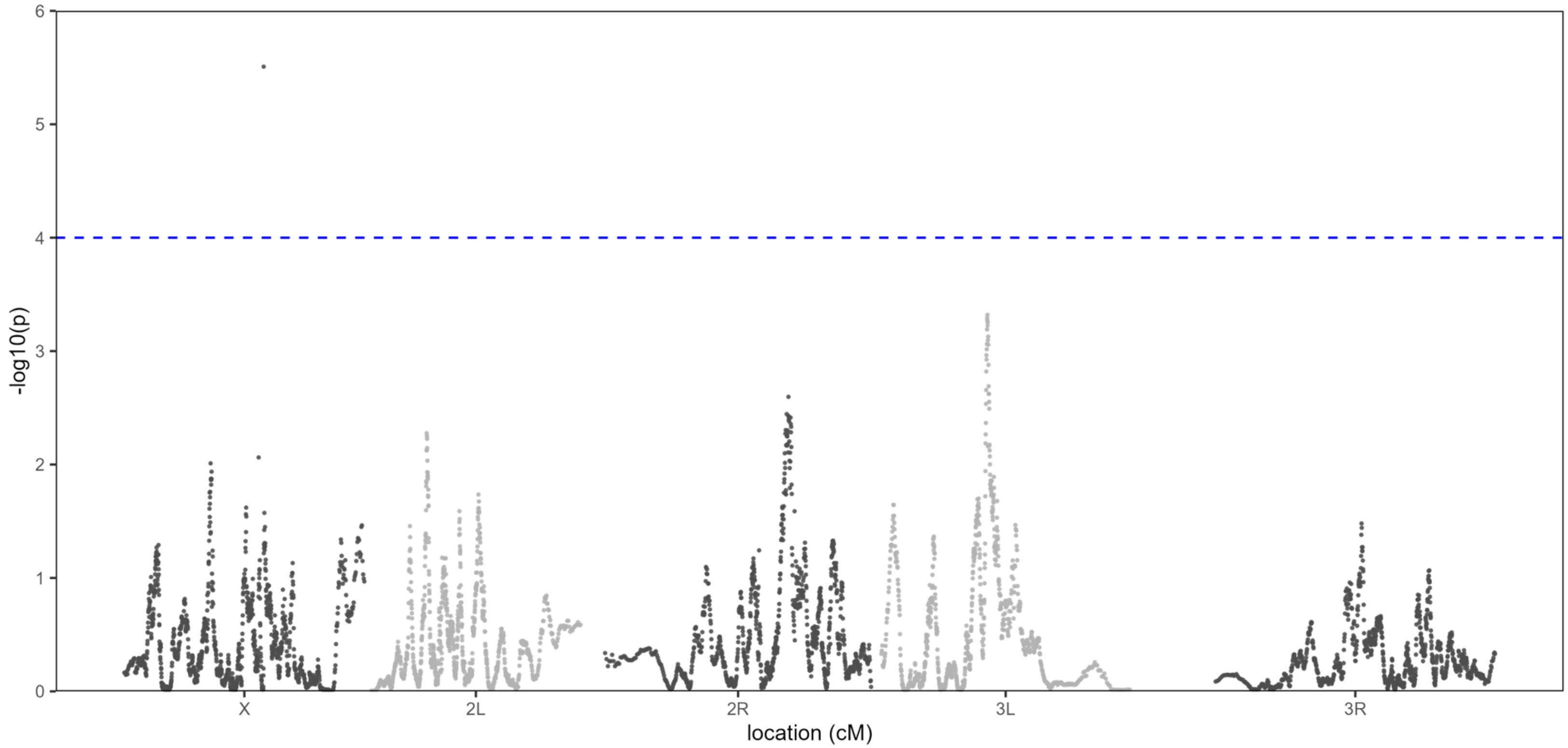

Supplementary Figure 2: Founder haplotype frequencies at mapped X-QTL.

Each of the 7 multi-panel figures on the next few pages depicts a single QTL (A-G) and comprises 4 panels. The top panel is identical to the relevant panel from Figure 2 in the main text, and shows the haplotype frequency difference (selection minus control) centered at each QTL. The middle panels show the estimated frequency for each founder haplotype separately for the control (left) and selected (right) populations through the QTL interval. The bottom panel shows the frequencies for each founder in the selected and control populations directly at the QTL peak. For QTL-A, the top panel shows the QTL is driven by A1 (carrying a resistance allele) and A7 (carrying a susceptibility allele). The remaining 6 founders show no/limited frequency change. The middle/bottom panels show that A7 and A1 are among the most common founders at this location in the genome. The remaining figures highlight a similar pattern for the other mapped QTL; QTL are driven by the more common founders at those genomic locations.

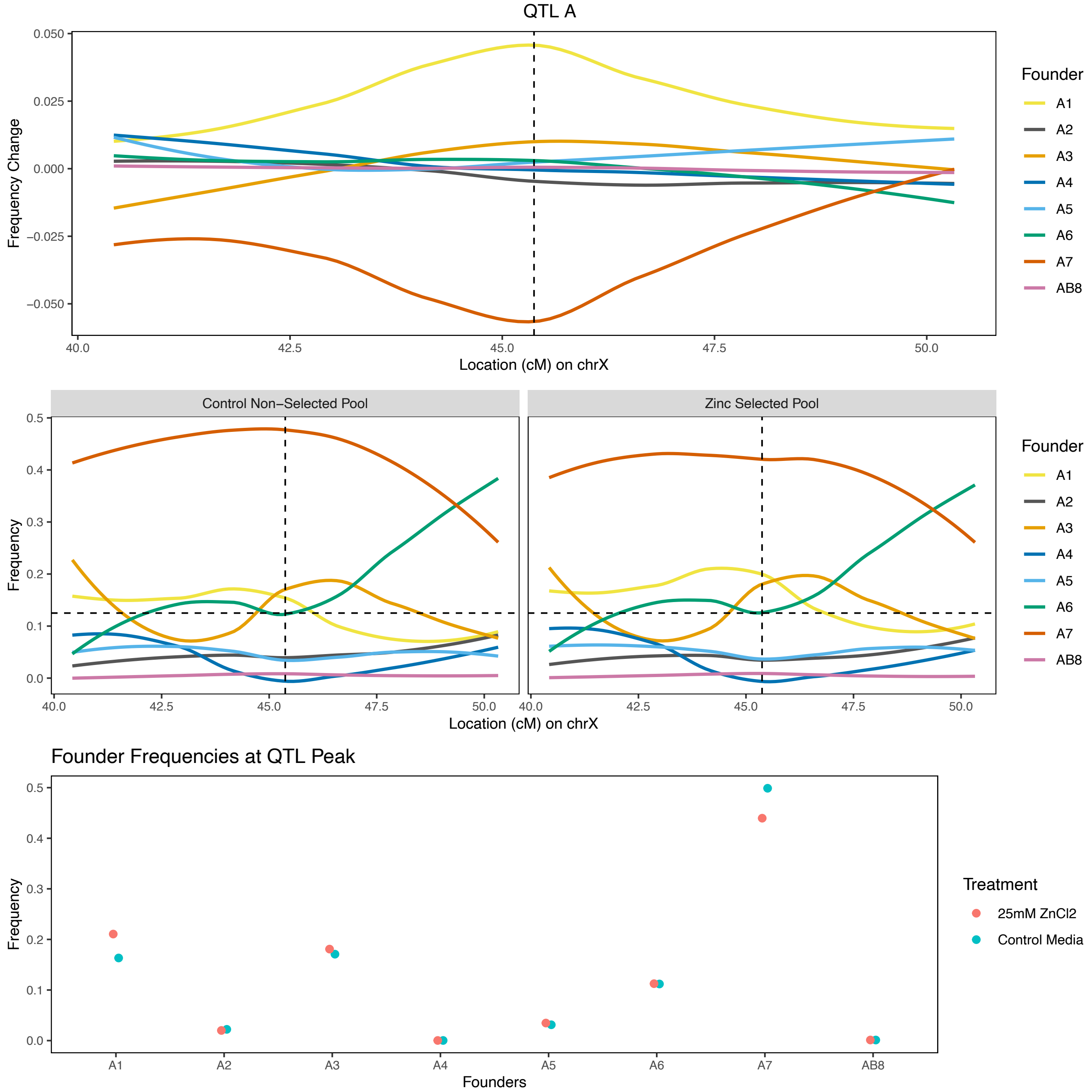

Supplementary Figure 2: Contd.

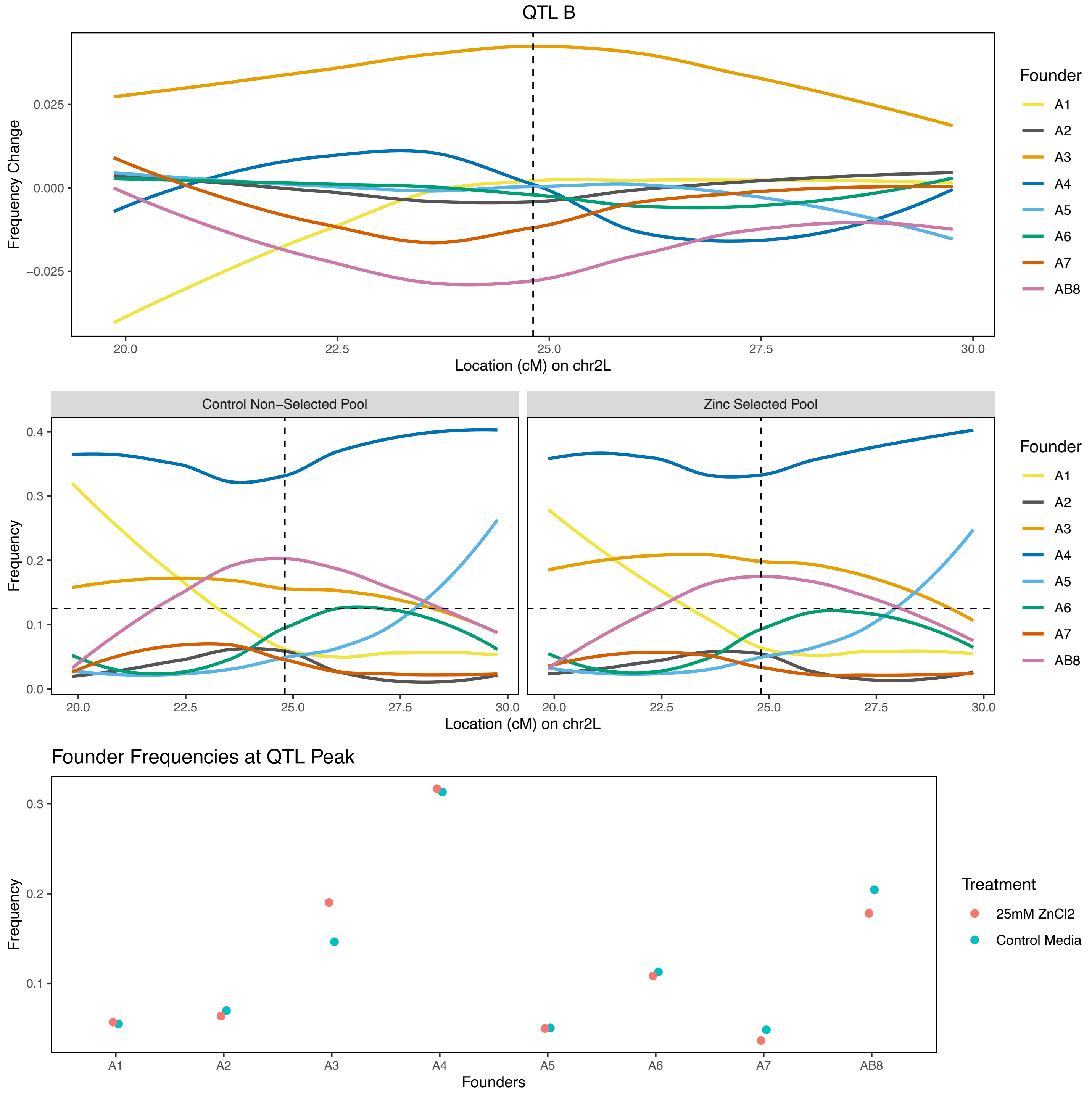

Supplementary Figure 2: Contd.

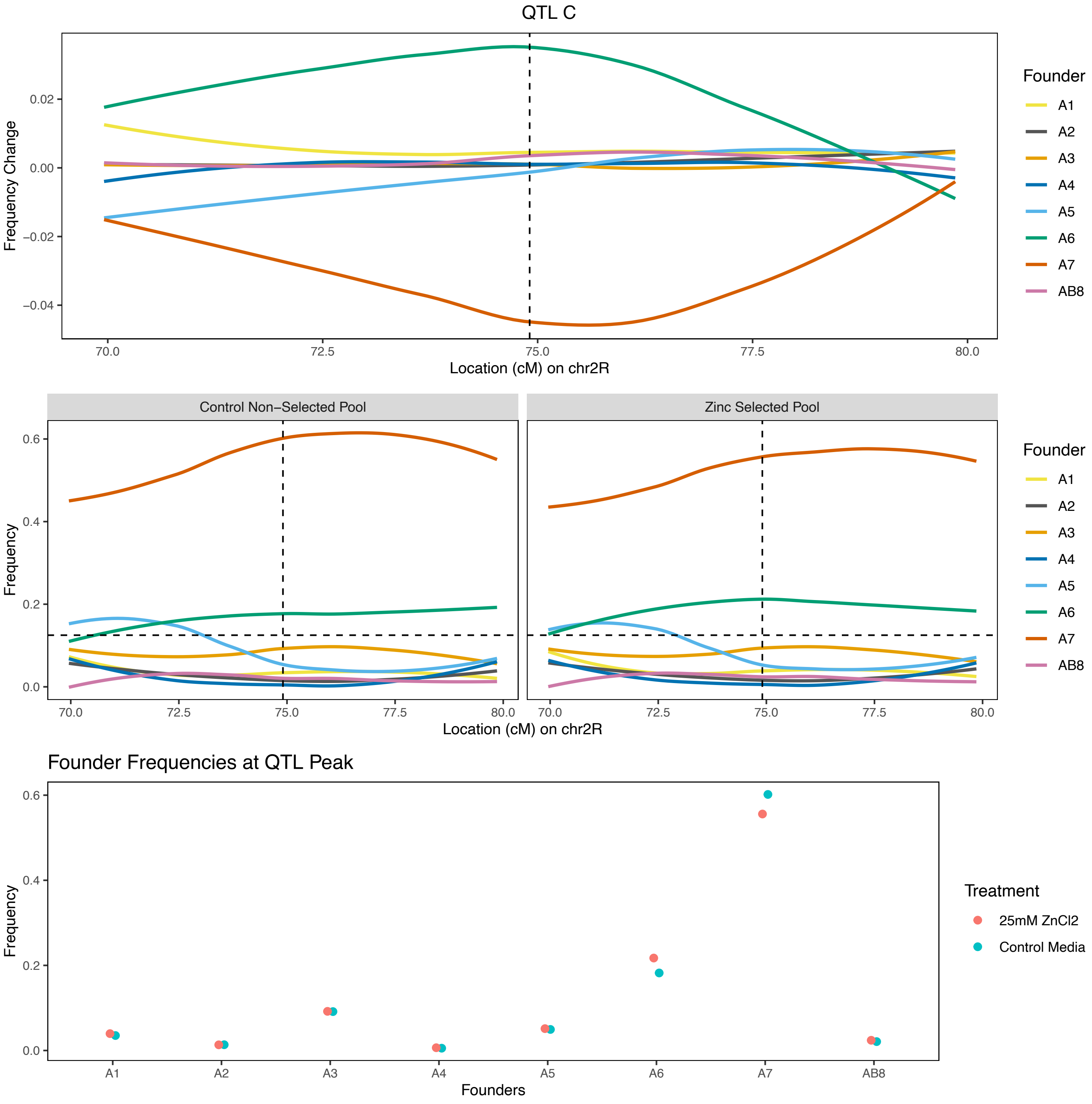

Supplementary Figure 2: Contd.

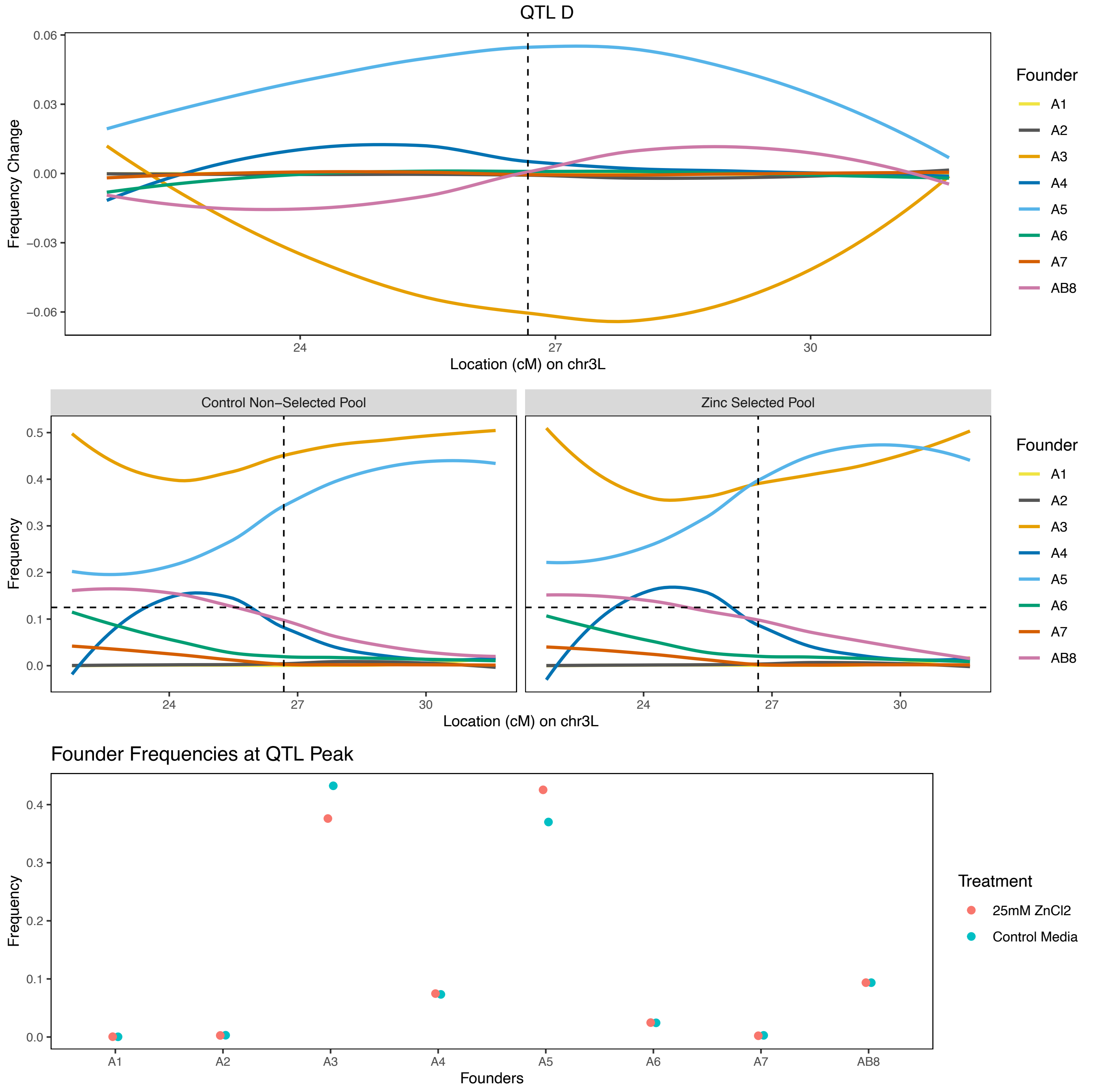

Supplementary Figure 2: Contd.

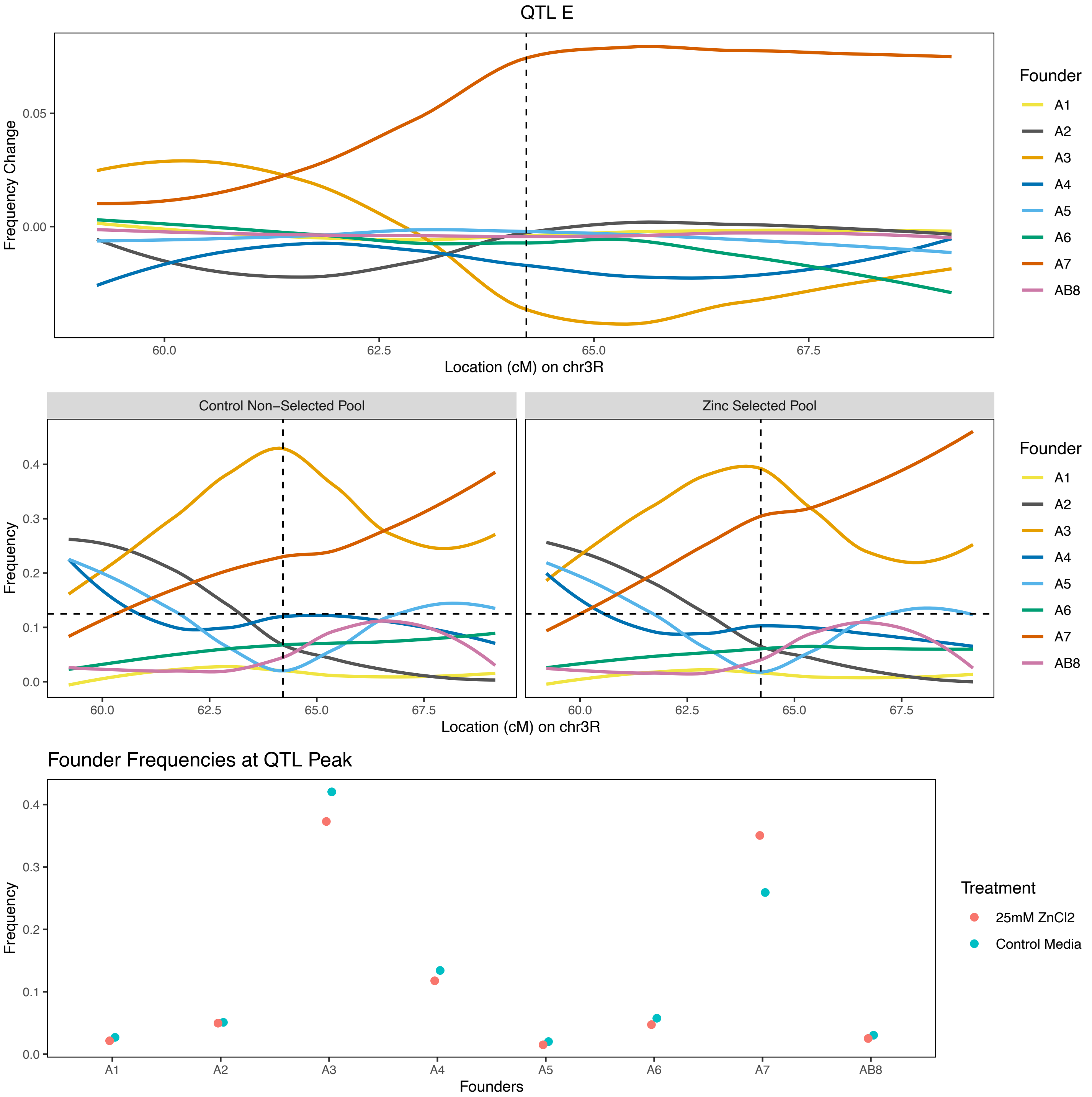

Supplementary Figure 2: Contd.

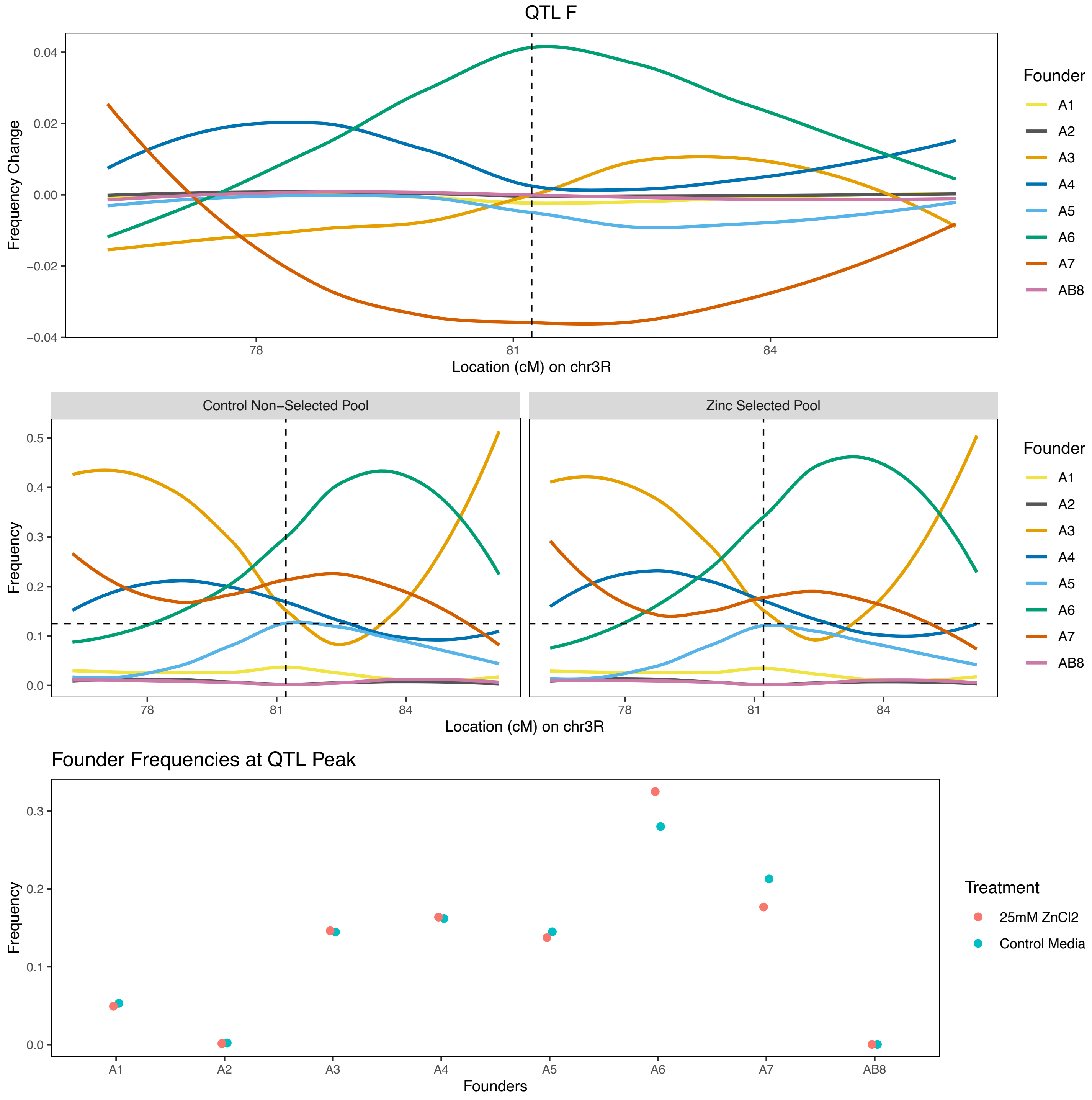

Supplementary Figure 2: Contd.

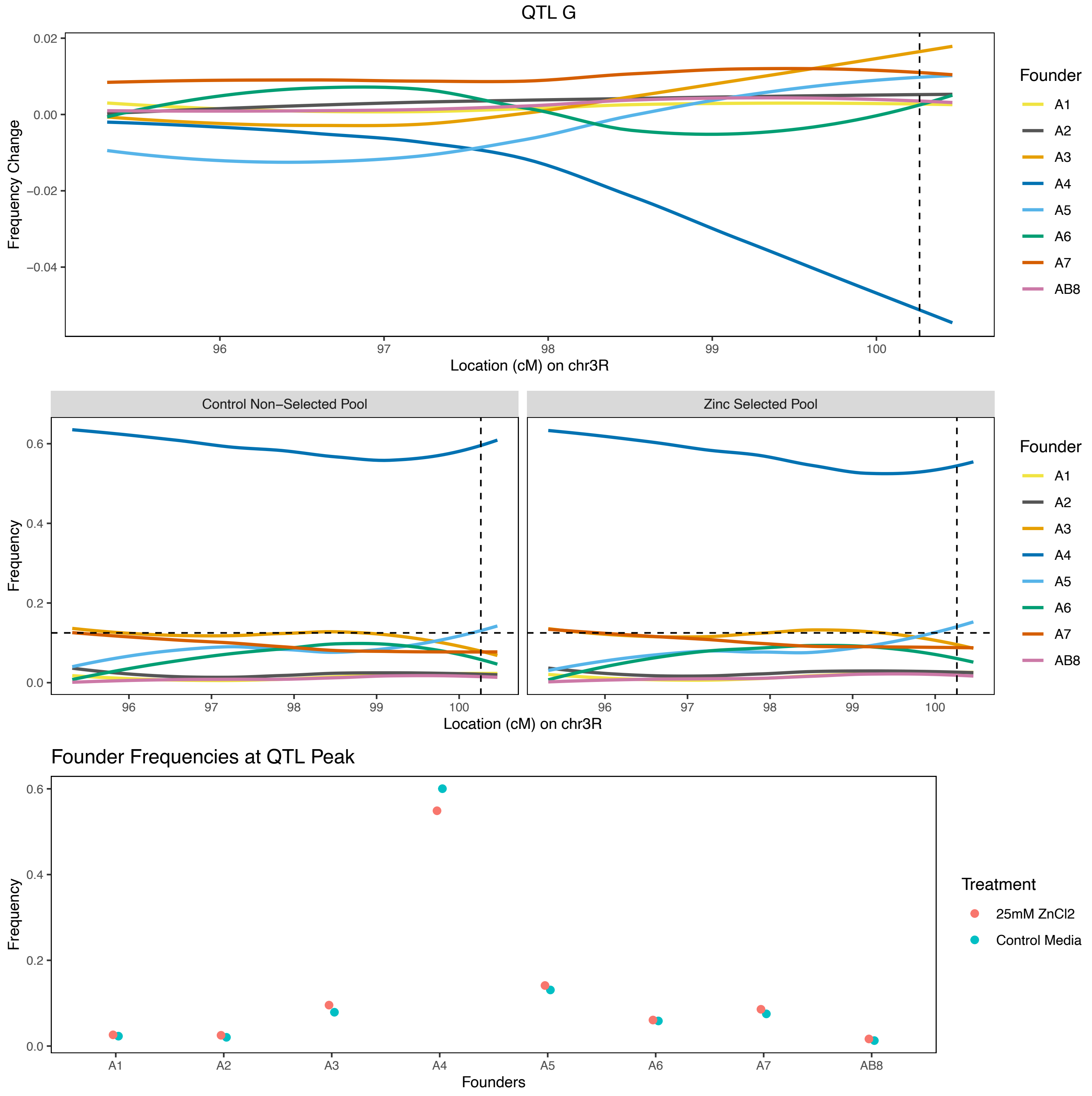

Supplementary Table 5: ANOVA tables for the emergence phenotype tested on several sets of animals derived from zinc-selected or control, un-selected populations. We do not consider sex as a factor when examining the emergence phenotype since we do not know the sex of the embryos that were placed in the test vials. Additionally, only the “X-QTL Subpopulation” was assayed over replicate batches, so replicate is not considered in the other two analyses. (Table is associated with Figure 3 in the main text.)

X-QTL Subpopulation Emergence in Water-control or Zinc-containing Treatments (Figure 3A)

Factor	Df	Sum Sq	Mean Sq	F value	Pr(>F)
Replicate	1	0.00035283	0.00035283	0.07967825	0.77908816
Population	1	0.062398	0.062398	14.0909992	0.00051774
Treatment	1	4.93959025	4.93959025	1115.48061	2.17E-32
Replicate:Population	1	0.00382413	0.00382413	0.8635825	0.35792577
Replicate:Treatment	1	0.00705382	0.00705382	1.5929253	0.21371151
Population:Treatment	1	0.0084848	0.0084848	1.91607577	**0.17343227**
Replicate:Population:Treatment	1	0.00491813	0.00491813	1.11063405	0.2978301
Residuals	43	0.19041333	0.00442822	nan	nan

Semi-inbred Line Emergence in Water-control or Zinc-containing Treatments (Figure 3B)

Factor	Df	Sum Sq	Mean Sq	F value	Pr(>F)
Population	1	0.02962922	0.02962922	1.06821751	0.30279943
Treatment	1	1.95817271	1.95817271	70.597684	1.59E-14
Population:Treatment	1	0.00079139	0.00079139	0.02853183	**0.86606302**
Residuals	172	4.77077558	0.02773707	nan	nan

Phenotyping-Only cohorts Emergence in Water-control or Zinc-containing Treatments (Figure 3C)

Factor	Df	Sum Sq	Mean Sq	F value	Pr(>F)
Population	1	0.04952116	0.04952116	8.73412322	0.00361778
Treatment	1	23.9417856	23.9417856	4222.64953	2.54E-113
Population:Treatment	1	0.03324203	0.03324203	5.86294704	**0.016633**
Residuals	153	0.86748691	0.00566985	nan	nan

Supplementary Table 6: ANOVA tables for the development time phenotype tested on several sets of animals derived from zinc-selected or control, un-selected populations. Only the "X-QTL Subpopulation" was assayed over replicate batches, so replicate is not considered in the other two analyses. (Associated with Figure 4.)

X-QTL Subpopulation Development Time in Water-control or Zinc-containing Treatments (Figure 4A)

Factor	Df	Sum Sq	Mean Sq	F value	Pr(>F)
Replicate	1	103.287902	103.287902	59.9639017	3.49E-14
Population	1	3.52536194	3.52536194	2.04665265	0.15299858
Treatment	1	4128.89214	4128.89214	2397.03275	1.42E-225
Sex	1	1.66583598	1.66583598	0.96710286	0.32575201
Replicate:Population	1	18.2856494	18.2856494	10.6157533	0.00117681
Replicate:Treatment	1	2.55154107	2.55154107	1.4812999	0.22399162
Population:Treatment	1	23.269981	23.269981	13.5094124	**0.00025599**
Replicate:Sex	1	1.32664343	1.32664343	0.77018426	0.38046865
Population:Sex	1	4.85406248	4.85406248	2.81803117	0.09366808
Treatment:Sex	1	0.72456241	0.72456241	0.42064548	0.51683313
Replicate:Population:Treatment	1	4.26393449	4.26393449	2.47543173	0.11610077
Replicate:Population:Sex	1	3.14926669	3.14926669	1.82831015	0.17677482
Replicate:Treatment:Sex	1	5.06302084	5.06302084	2.93934216	0.08689959
Population:Treatment:Sex	1	6.22343735	6.22343735	3.61302321	0.05774953
Replicate:Population:Treatment:Sex	1	13.6957354	13.6957354	7.95107387	0.0049452
Residuals	683	1176.46842	1.72250135	nan	nan

Semi-inbred Line Development Time in Water-control or Zinc-containing Treatments (Figure 4B)

Factor	Df	Sum Sq	Mean Sq	F value	Pr(>F)
Population	1	892.791338	892.791338	245.946935	1.81E-53
Treatment	1	20130.7982	20130.7982	5545.64981	0
Sex	1	13.0415602	13.0415602	3.59270036	0.0581234
Population:Treatment	1	411.081608	411.081608	113.245119	**5.14E-26**
Population:Sex	1	1.78484459	1.78484459	0.49169054	0.48322486
Treatment:Sex	1	1.28305092	1.28305092	0.35345599	0.55220507
Population:Treatment:Sex	1	0.77520082	0.77520082	0.213553	0.64402798
Residuals	3218	11681.3919	3.63001611	nan	nan

Phenotyping-Only cohorts Development Time in Water-control or Zinc-containing Treatments (Figure 4C)

Factor	Df	Sum Sq	Mean Sq	F value	Pr(>F)
Population	1	5.24647536	5.24647536	1.76834605	0.18366898
Treatment	1	16004.7828	16004.7828	5394.47773	0
Sex	1	20.7637731	20.7637731	6.99851493	0.00819229
Population:Treatment	1	2.66621997	2.66621997	0.89866038	**0.34320316**
Population:Sex	1	4.62242874	4.62242874	1.55800858	0.21203613
Treatment:Sex	1	36.2104188	36.2104188	12.2048702	0.00048224
Population:Treatment:Sex	1	0.06832184	0.06832184	0.02302816	0.87939216
Residuals	3695	10962.6317	2.96688273	nan	nan

Supplementary Figure 3: Variation in phenotype among RNAi control genotypes.

As controls for RNAi knockdowns we examined emergence and development time for 4 different controls; UAS-GFP, UAS-Luciferase, and strains that carry empty attP2 or attP40 docking sites. Parental inbred control strains were crossed to the *mex1*-GAL4 strain, and the F1 genotypes (e.g., *mex1*-GAL4:UAS-GFP) were tested on either water control or zinc-containing media. Notably, *mex1*-GAL4:attP2 (highlighted in blue in the figure) exhibited substantially lower emergence in the $ZnCl_2$ treatment (left panel), and was the slowest developing of all 4 control genotypes on both media types (right panel). See also Supplementary Table 7. Note the inverted *y*-axis used for the development time (right) panel. This was done such that a visibly negative slope for both emergence *and* development time yielded the same inference; zinc treatment led to a worse outcome (reduced emergence or longer development time.) Points are phenotype means for a given genotype/treatment, and vertical bars are 95% confidence intervals.

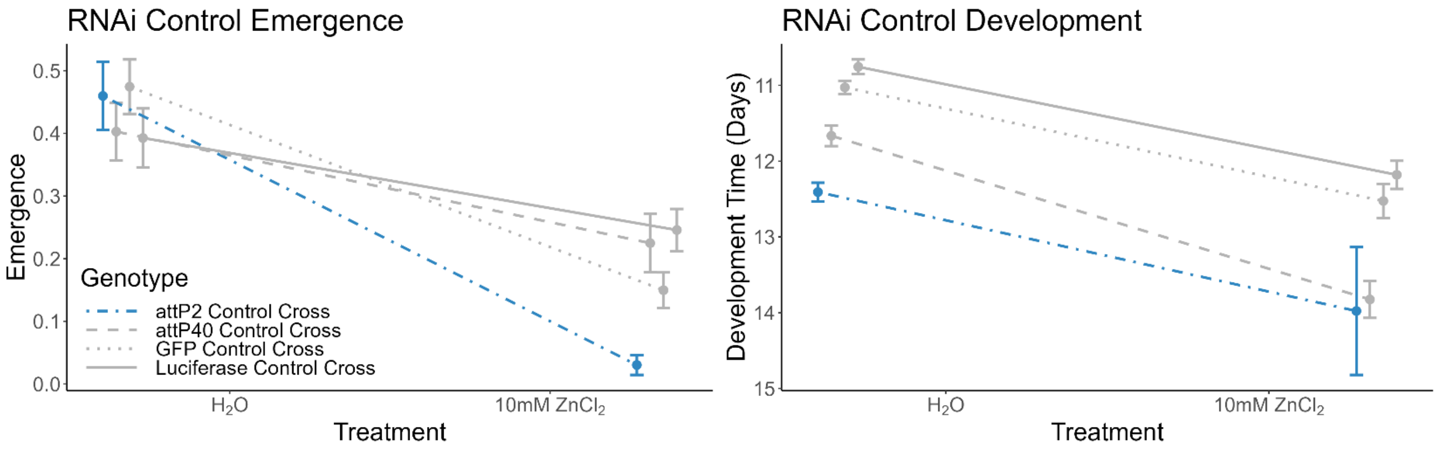

Supplementary Table 7: Summary of variation in phenotype among RNAi control genotypes.

Control Genotype *	Water Control Treatment				10mM $ZnCl_2$			
	Total Vials **	Total Flies Emerged	Average Emergence	Average Development Time	Total Vials **	Total Flies Emerged	Average Emergence	Average Development Time
attP2	29	665	0.460	**12.4**	29	44	**0.030**	**14.0**
attP40	29	580	0.402	11.7	29	326	0.225	13.8
UAS-GFP	29	686	0.474	11.0	29	217	0.150	12.5
UAS-Luc	29	567	0.393	10.8	28	344	0.246	12.2

* Genotypes tested are F1 heterozygotes between the *mex1*-GAL4 driver strain and the listed UAS/control strain.

** Each vial initially contains 50 eggs.

Supplementary Table 8: Full ANOVA tables for each comparison between an RNAi knockdown genotype and each genetic control for both emergence and development time. Of interest for each test is the significance of the genotype-by-treatment interaction, since this reflects any difference between knockdown and controls in their response to zinc treatment. In the table the “Phenotype” column indicates whether the test is for emergence or development time, “Control” indicates which genetic control the RNAi knockdown is compared to, “Gene_KD_UAS_Line” gives the gene ID and the Bloomington *Drosophila* Stock Center (BDSC) ID for the UAS-RNAi strain used, and “UAS_Chromosome” defines which chromosome (chromosome 2 or 3) the UAS-RNAi transgene resides on. Note that due to very low numbers of animals for certain genotype/treatment/sex combinations some coefficients could not be estimated, and not all possible interactions were tested. (Associated with Supplementary Figure 4 and Table 2.)

*Due to its length, Supplementary Table 8 is available for separate download as a *.csv file.*

Supplementary Figure 4: Effects of RNAi expression knockdown on emergence and development time.

Each of the 18 figures on the next few pages focuses on a single gene, and has 2 panels; the right panel shows the fraction of egg-to-adult emergence, the left panel highlights development time. Note the inverted *y*-axis used for the development time (right) panel. This was done such that a visibly negative slope for both emergence *and* development time yields the same inference; zinc treatment leads to a worse outcome (reduced emergence or longer development time.) Each of the plots shows the exact same data for the 4 control genotypes (*mex1*-GAL4:attP2, *mex1*-GAL4:attP40, *mex1*-GAL4:UAS-GFP, and *mex1*-GAL4:UAS-Luc) in various gray lines, along with the data for *mex1*-GAL4:UAS-RNAi genotype targeting a specific gene (in red or blue; when we targeted the same gene with two different RNAi transgenes, both are shown on the same plot). Points are means across all vials measured per genotype per treatment, while vertical bars are 95% confidence intervals. Our analyses are focused on identifying significant genotype-by-treatment effects of RNAi knockdown (i.e., is the effect of treatment different for the RNAi knockdown and genetic control genotypes), which is seen in these plots via a different slope. See in-text Table 2 for a summary of the statistical analyses, and Supplementary Table 8 for full details of the ANOVAs.

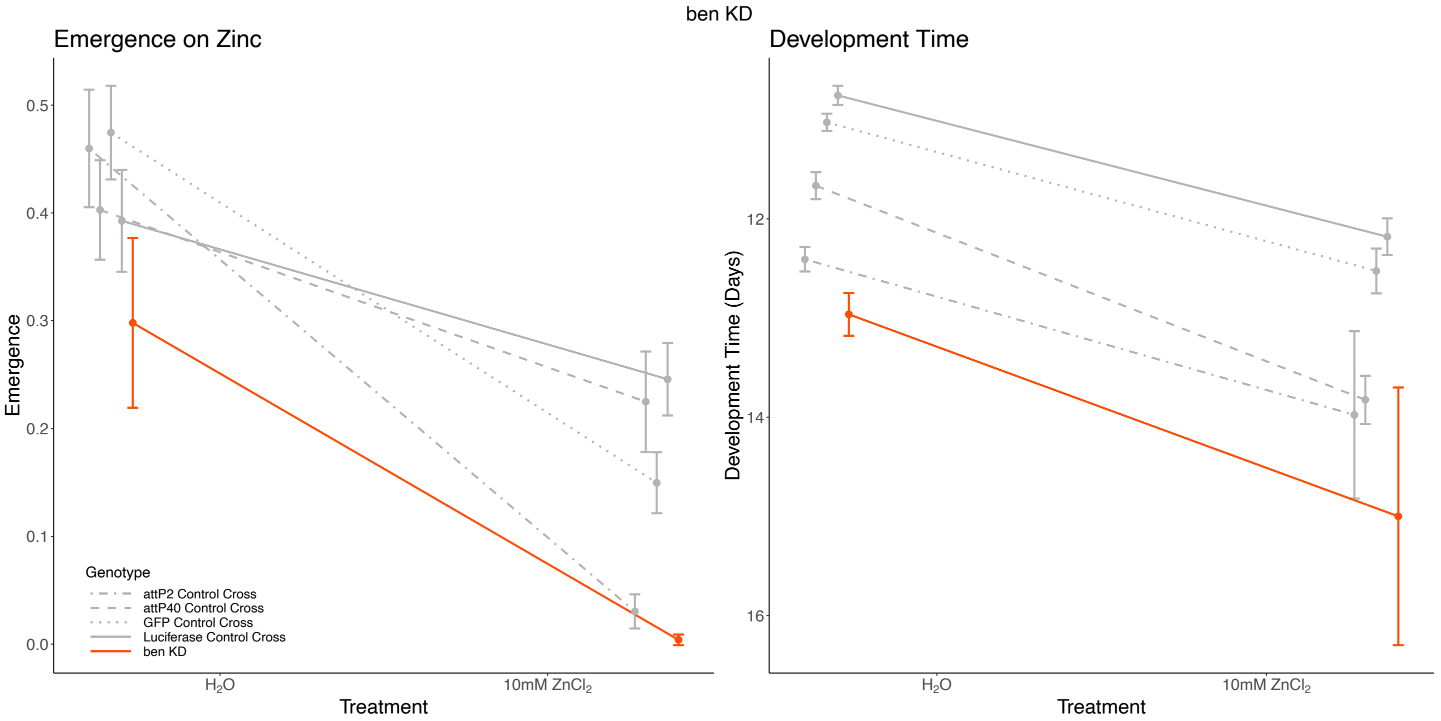

Supplementary Figure 4: Contd.

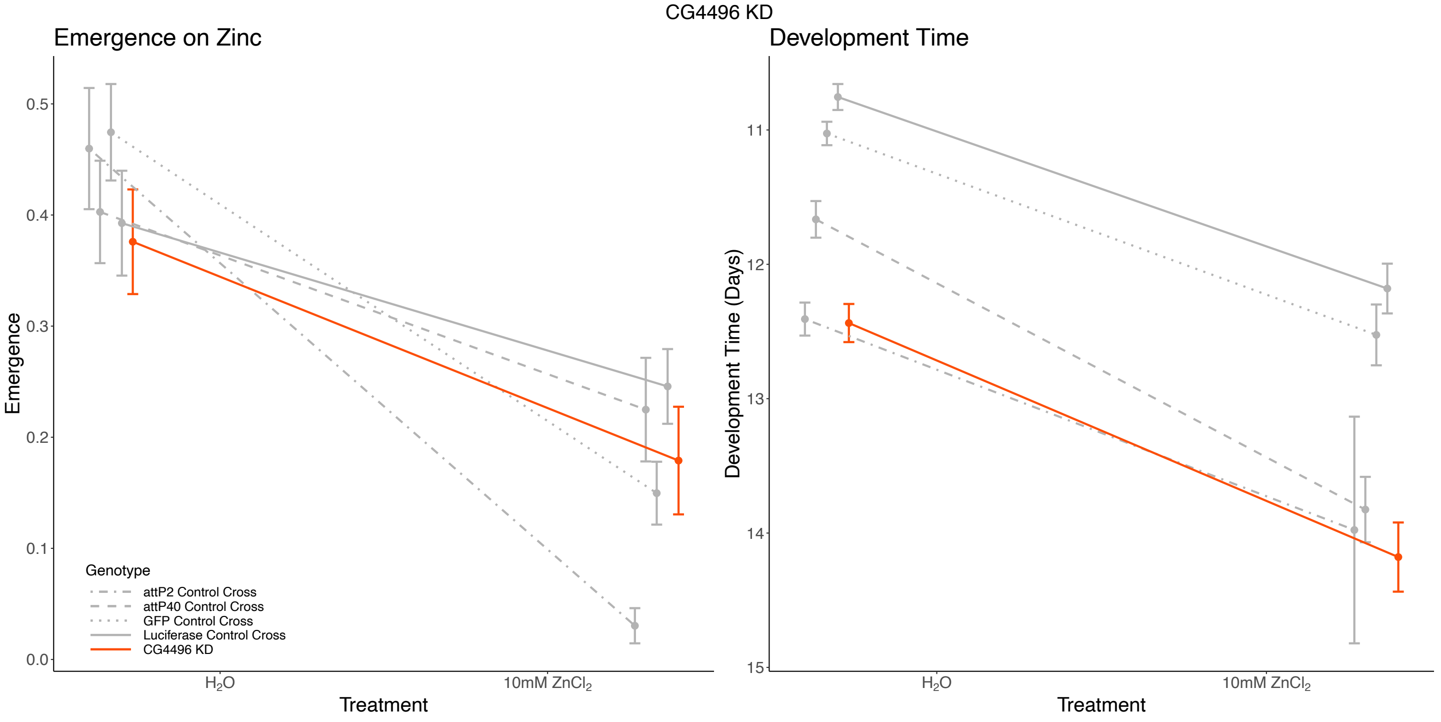

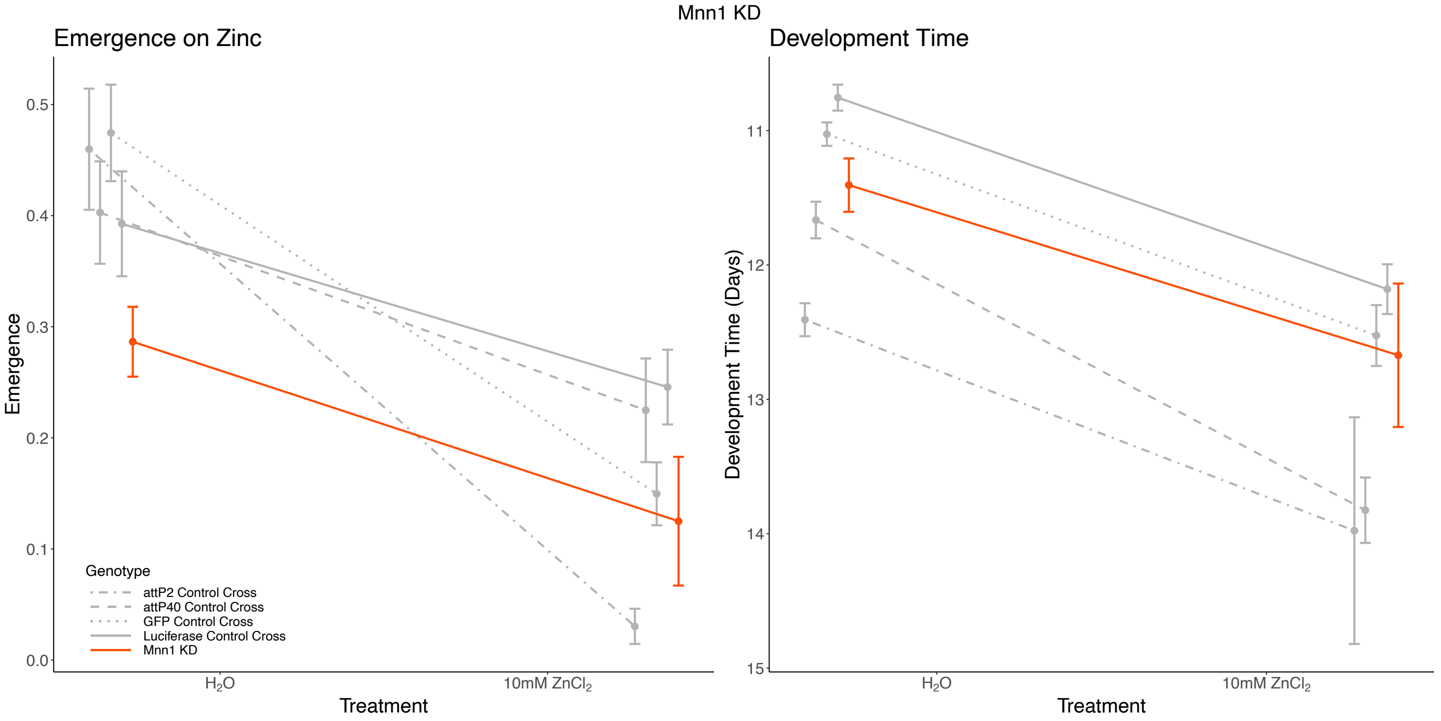

Supplementary Figure 4: Contd.

Ndae1 KD

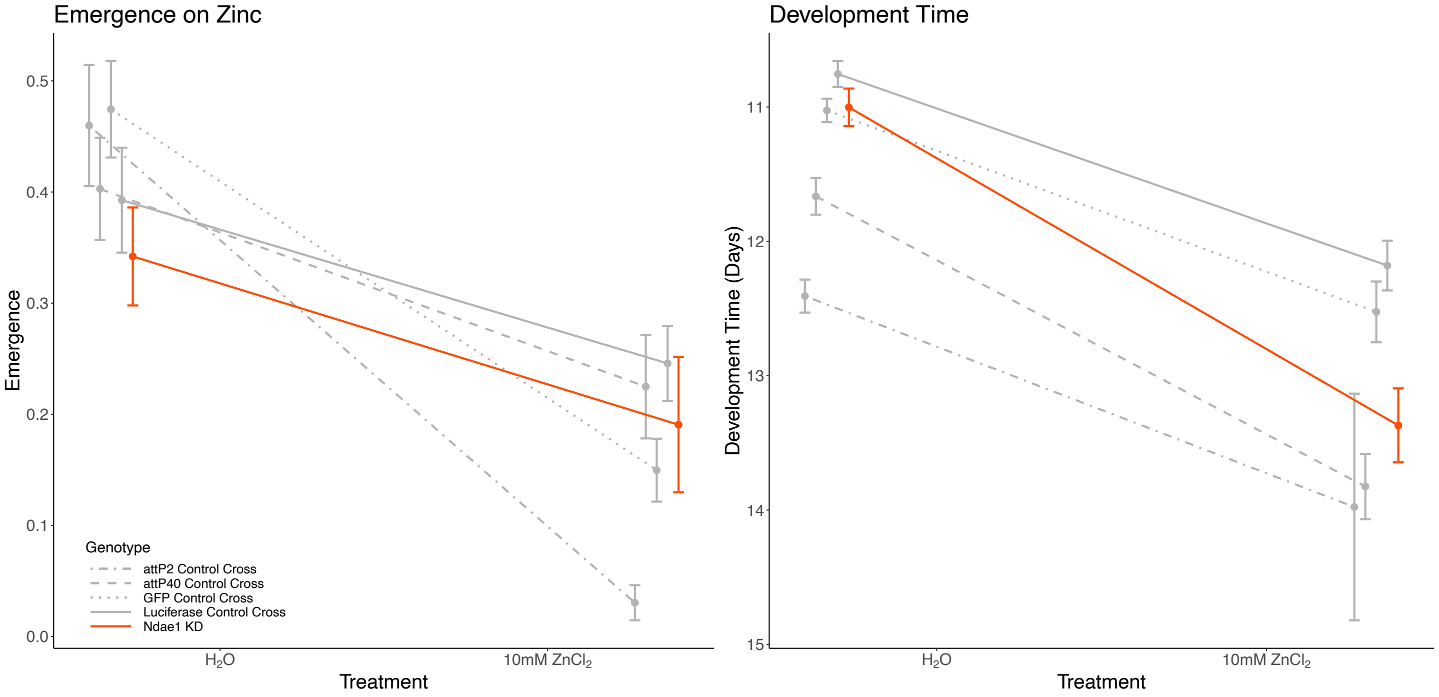

Trpm KD

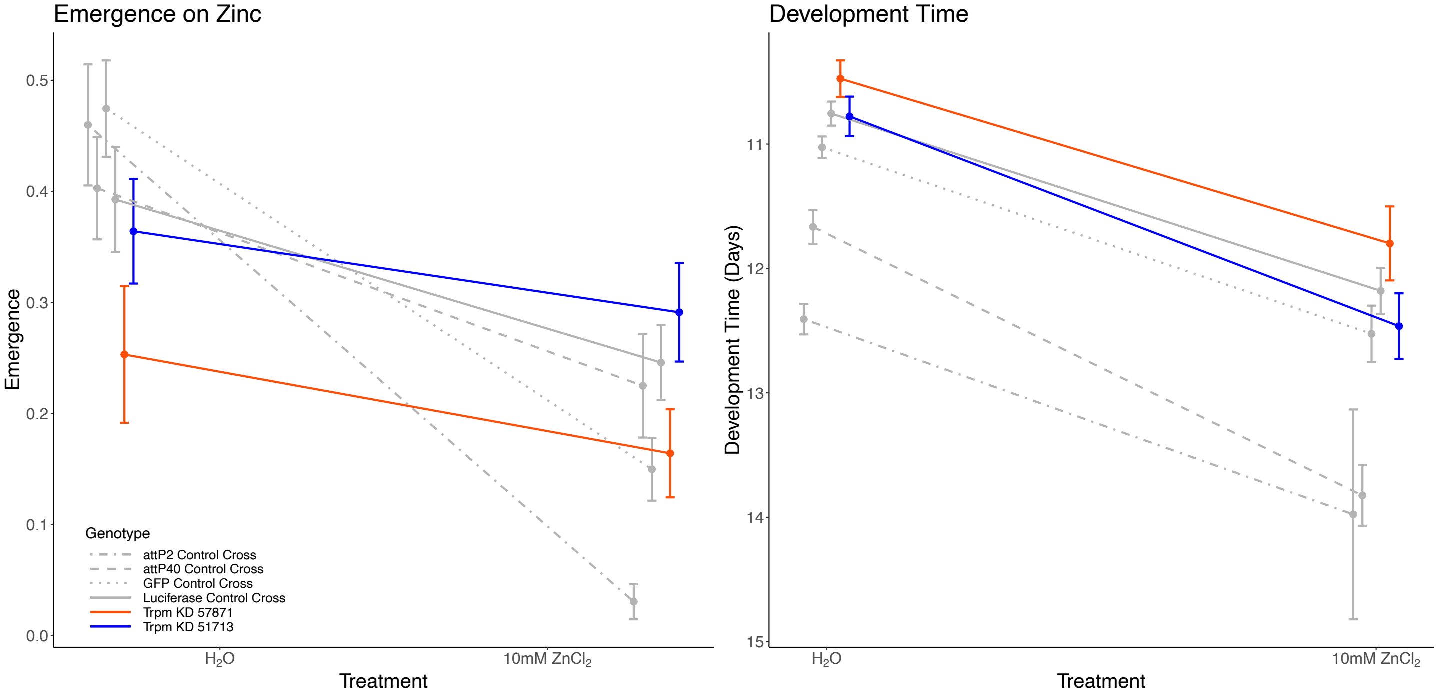

Supplementary Figure 4: Contd.

GluRIB KD

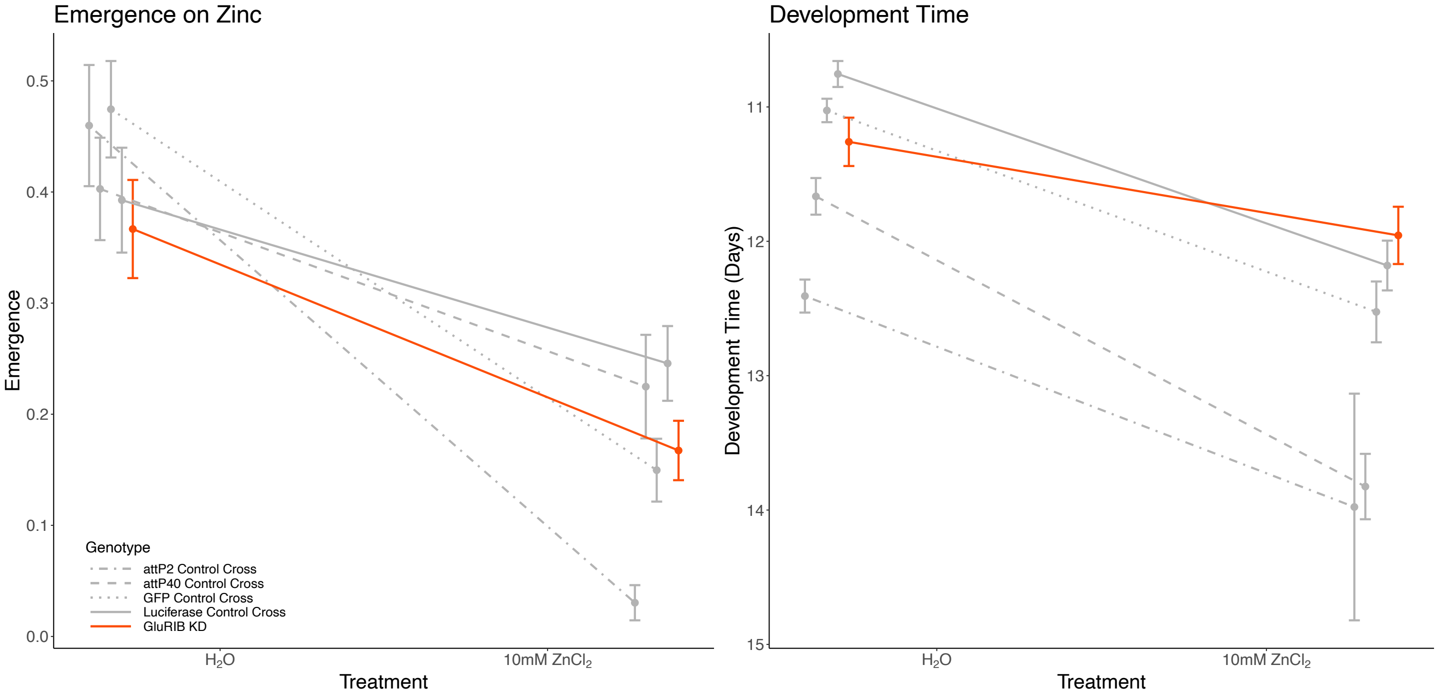

Hsp22 KD

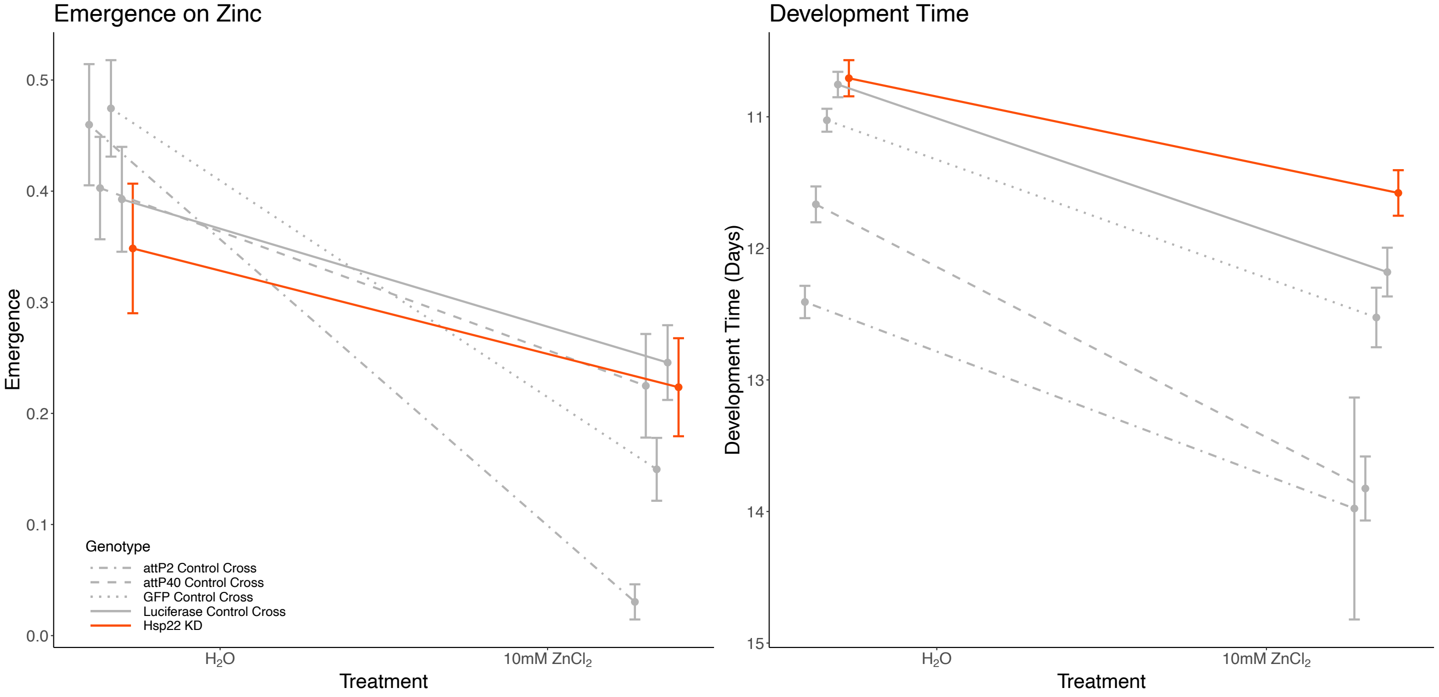

Supplementary Figure 4: Contd.

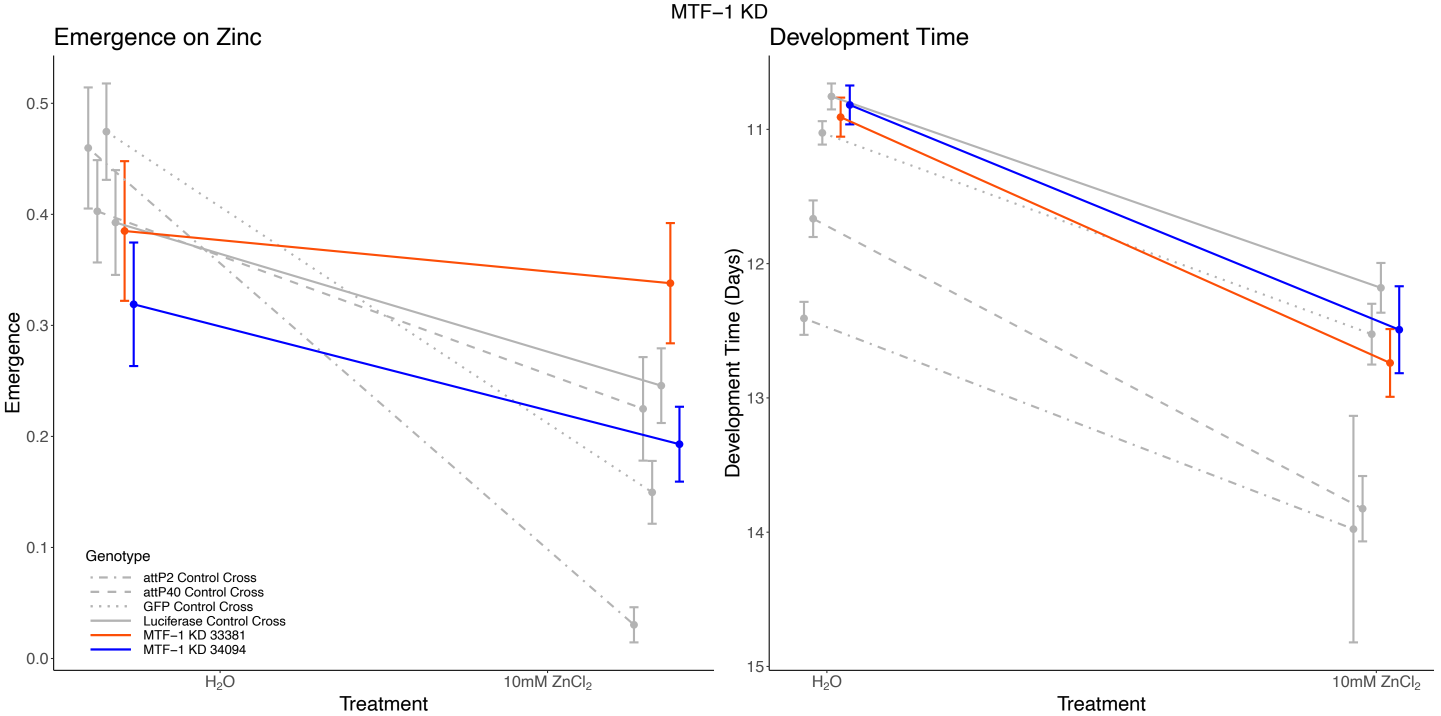

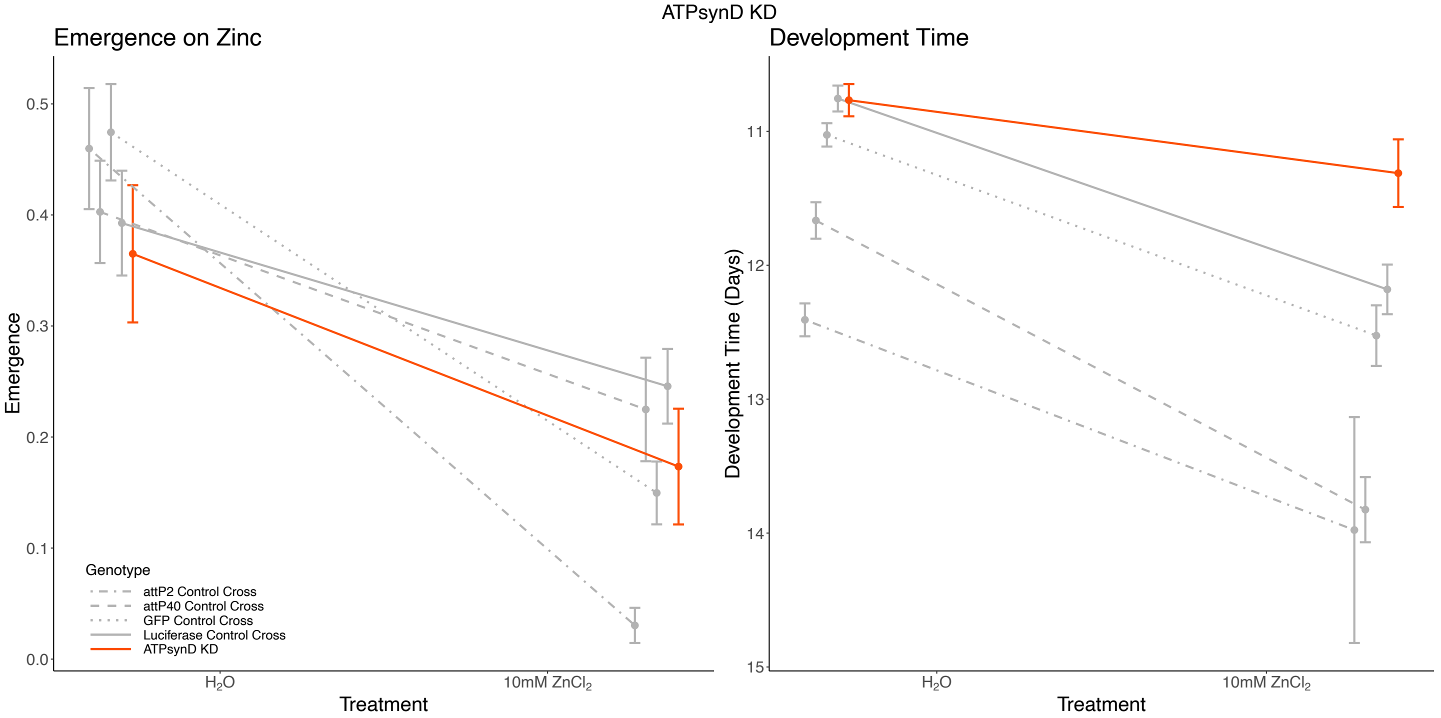

Supplementary Figure 4: Contd.

Mekk1 KD

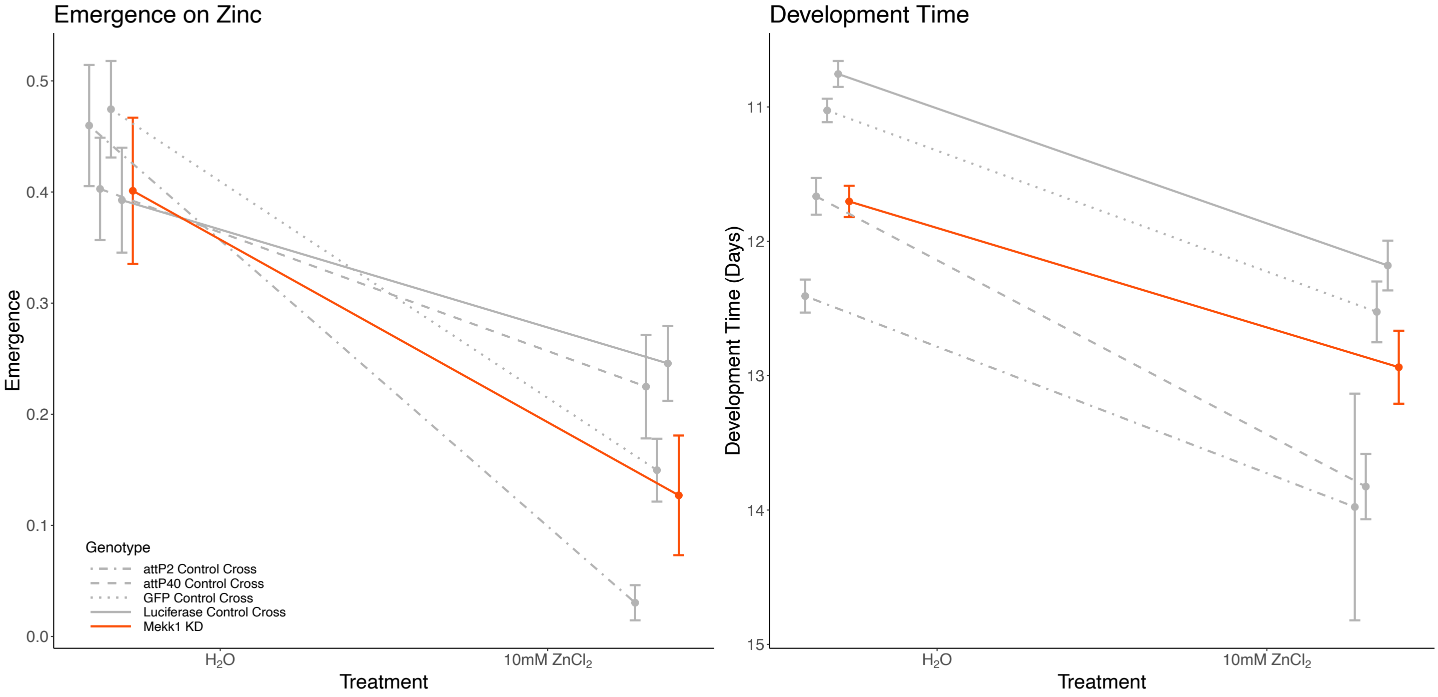

Octa2R KD

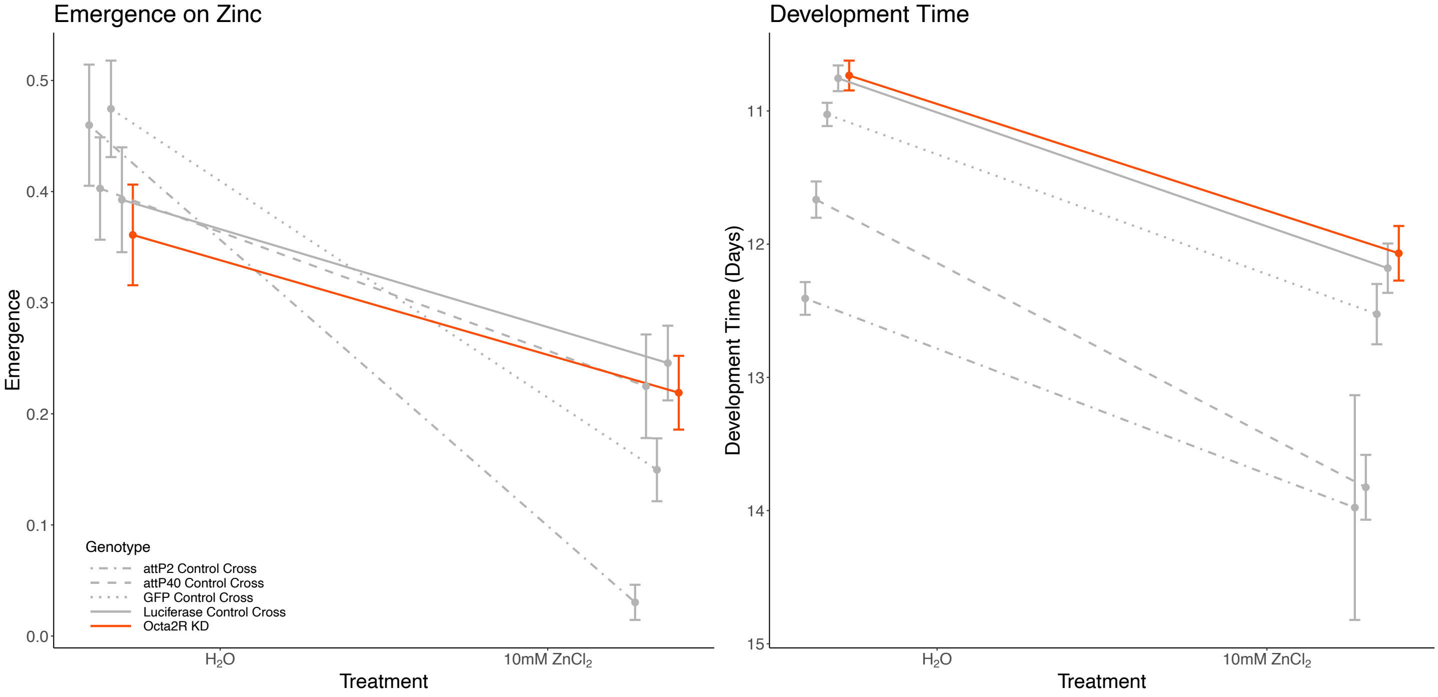

Supplementary Figure 4: Contd.

Xrp1 KD

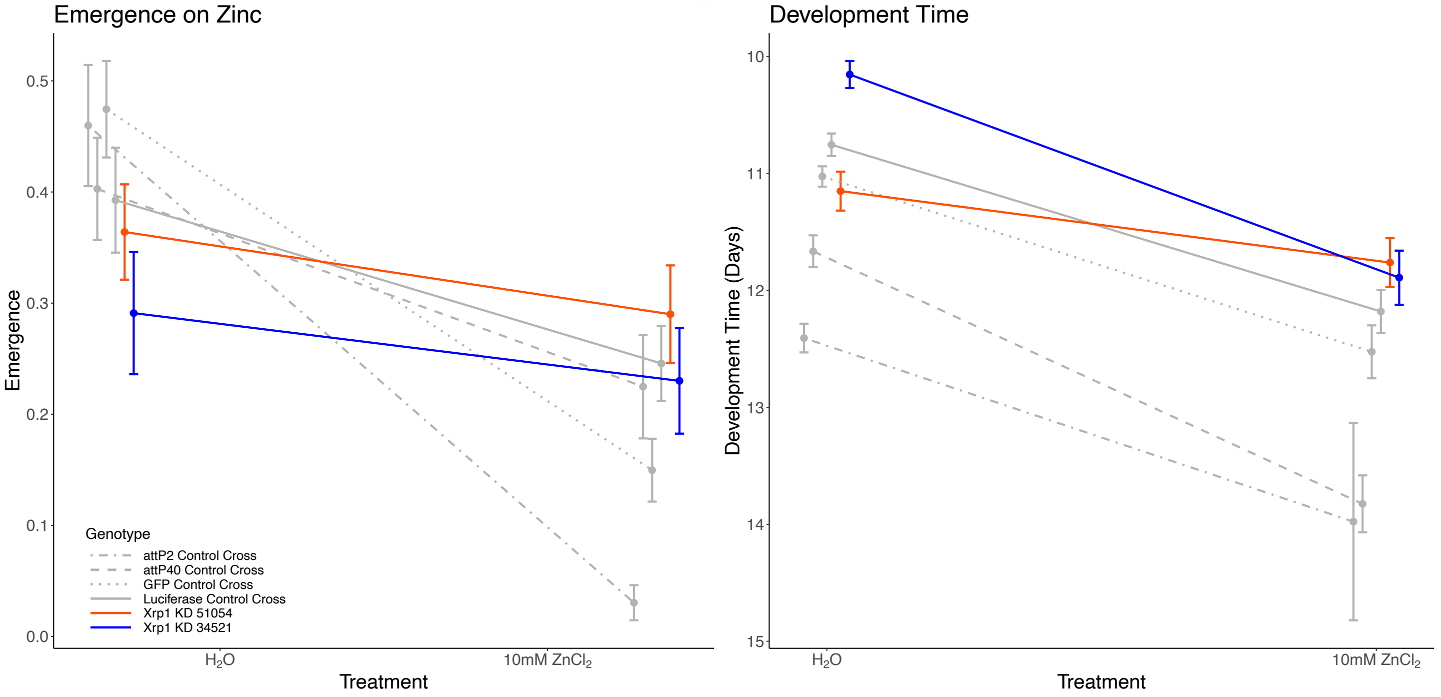

Ndc1 KD

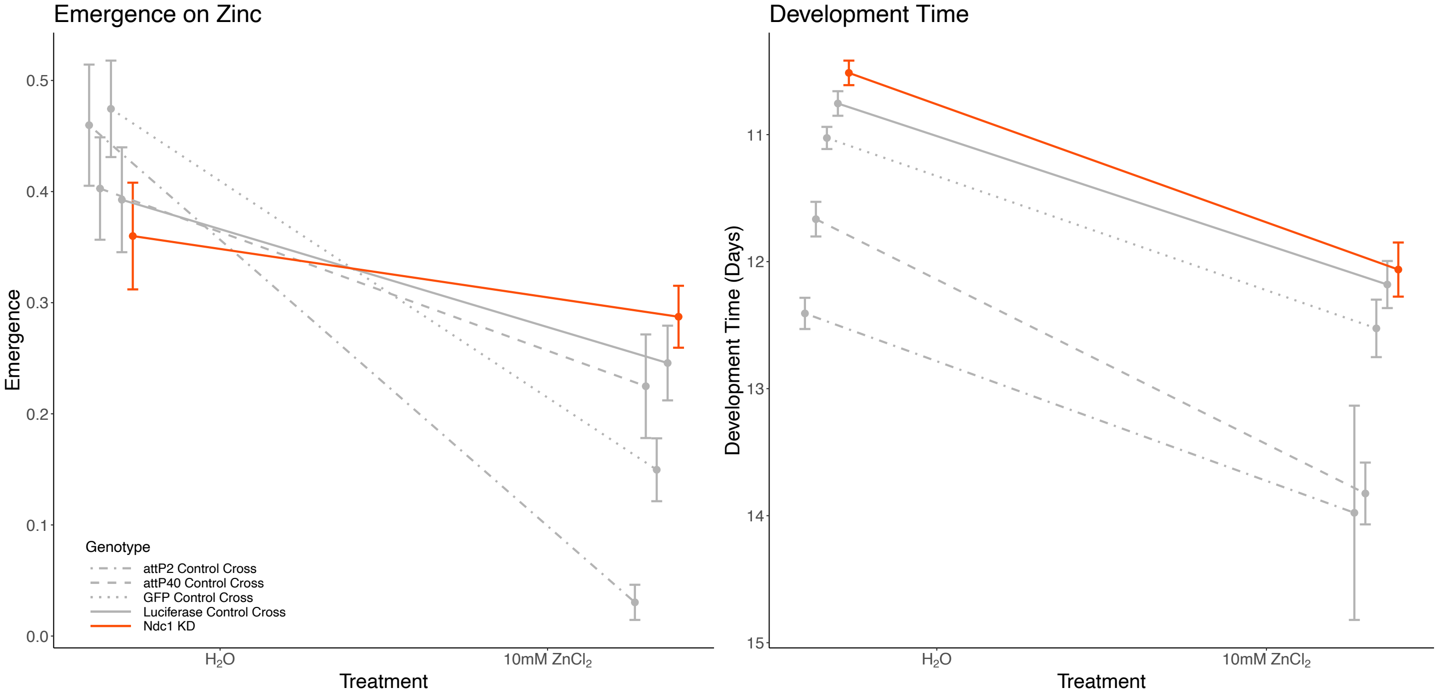

Supplementary Figure 4: Contd.

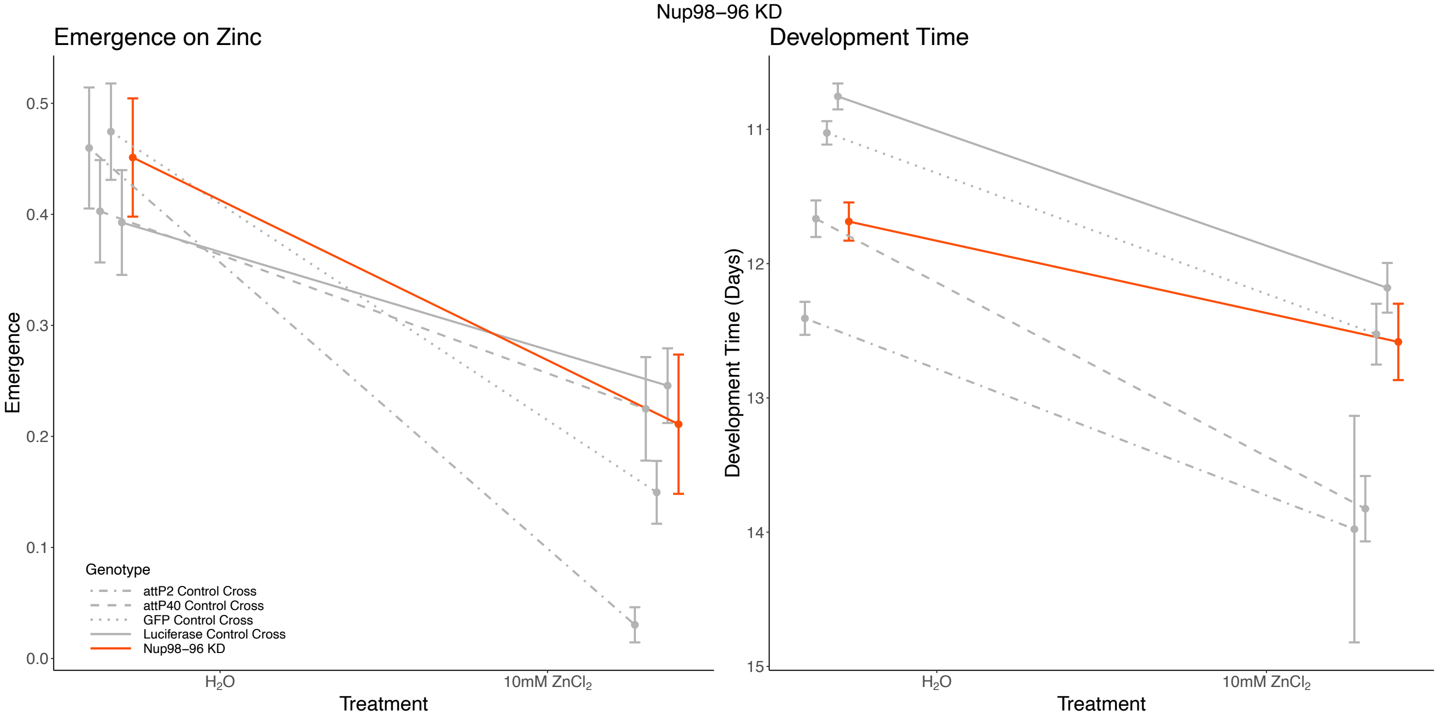

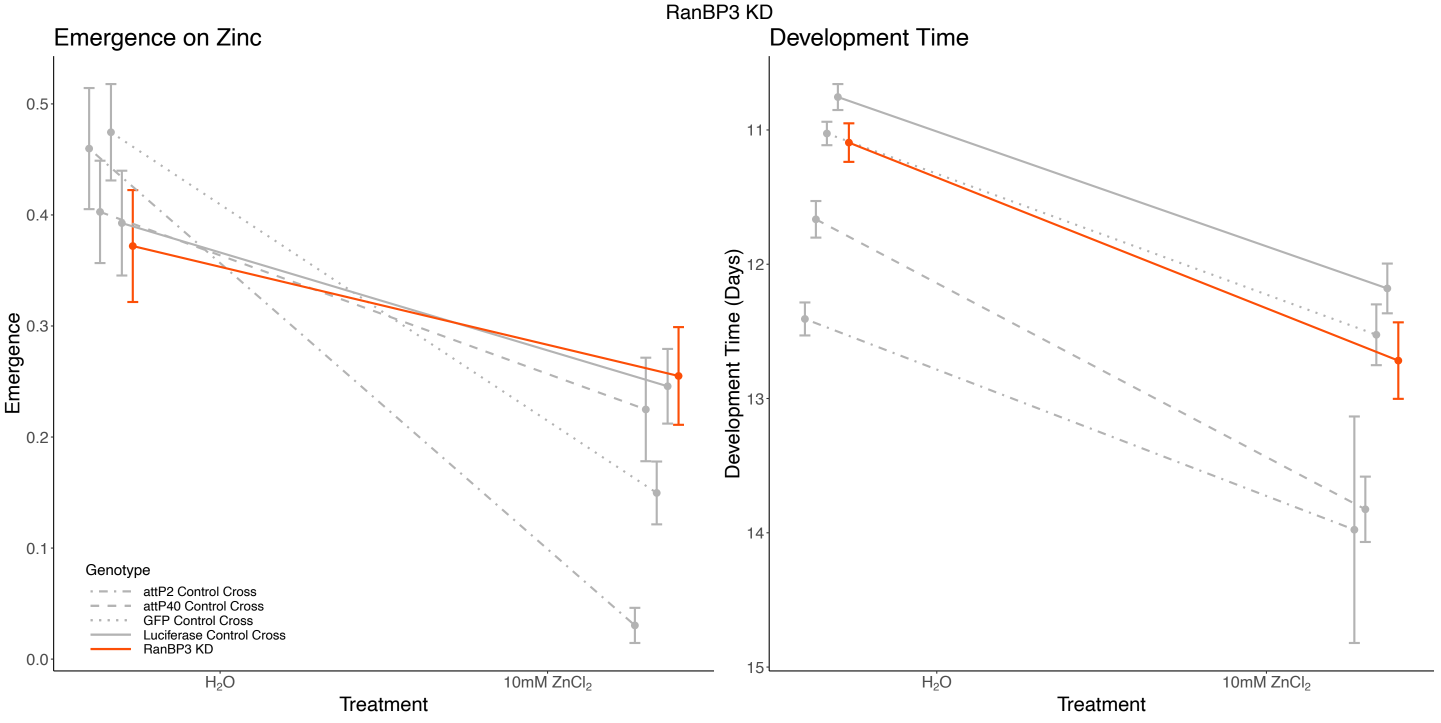

Supplementary Figure 4: Contd.

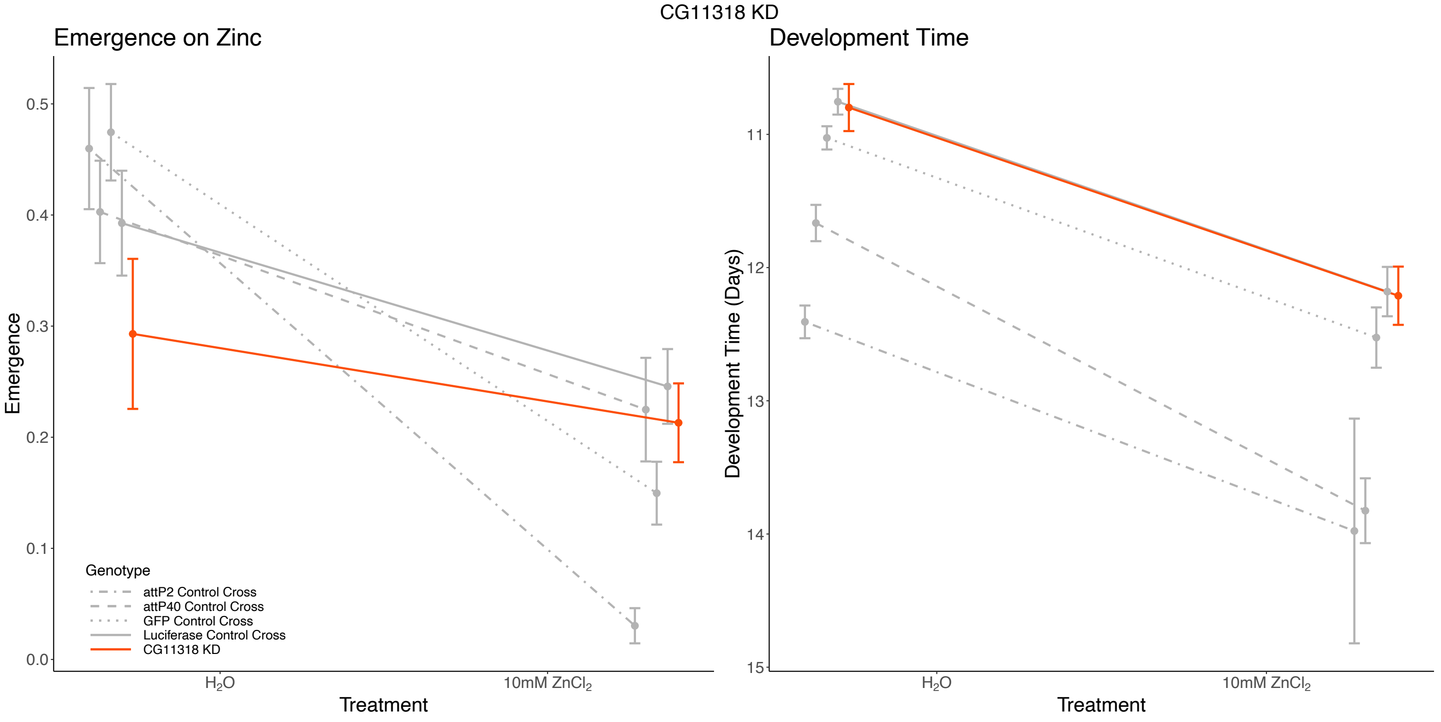

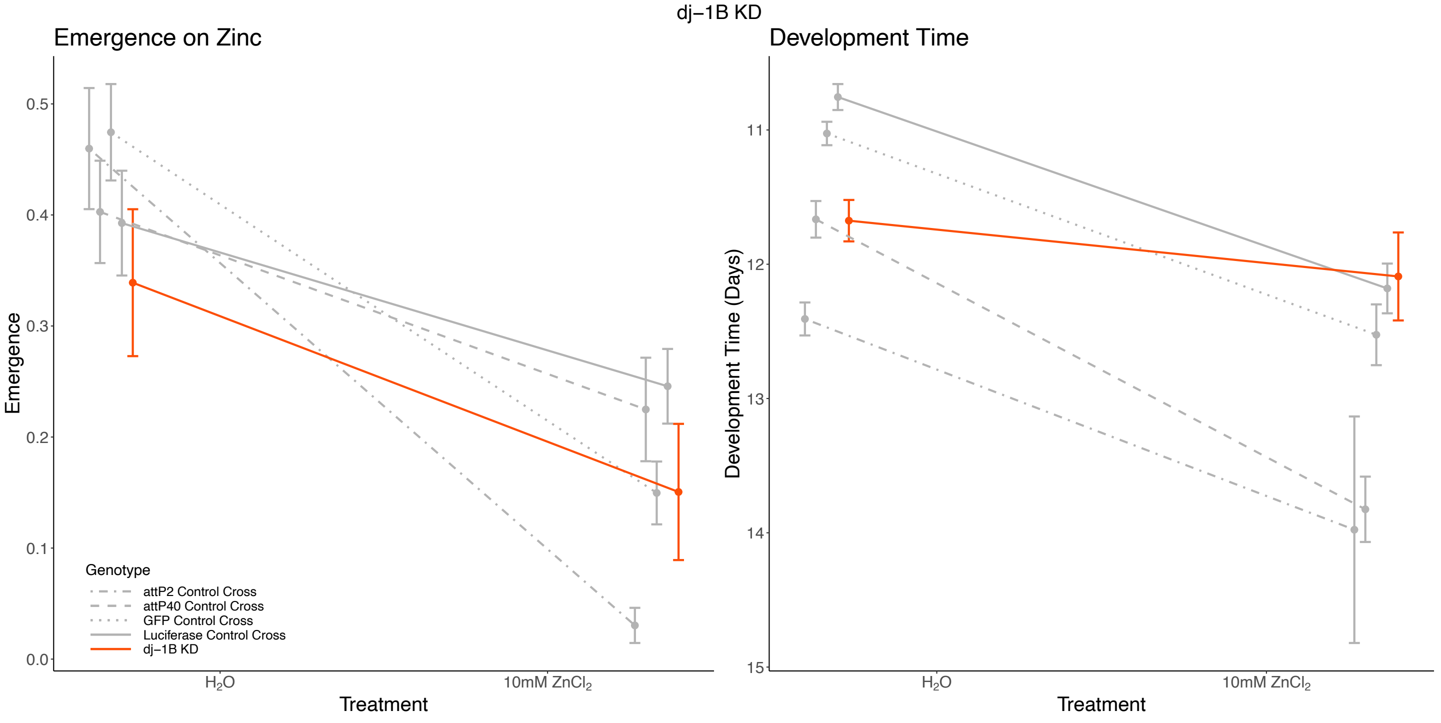

Supplementary Figure 4: Contd.

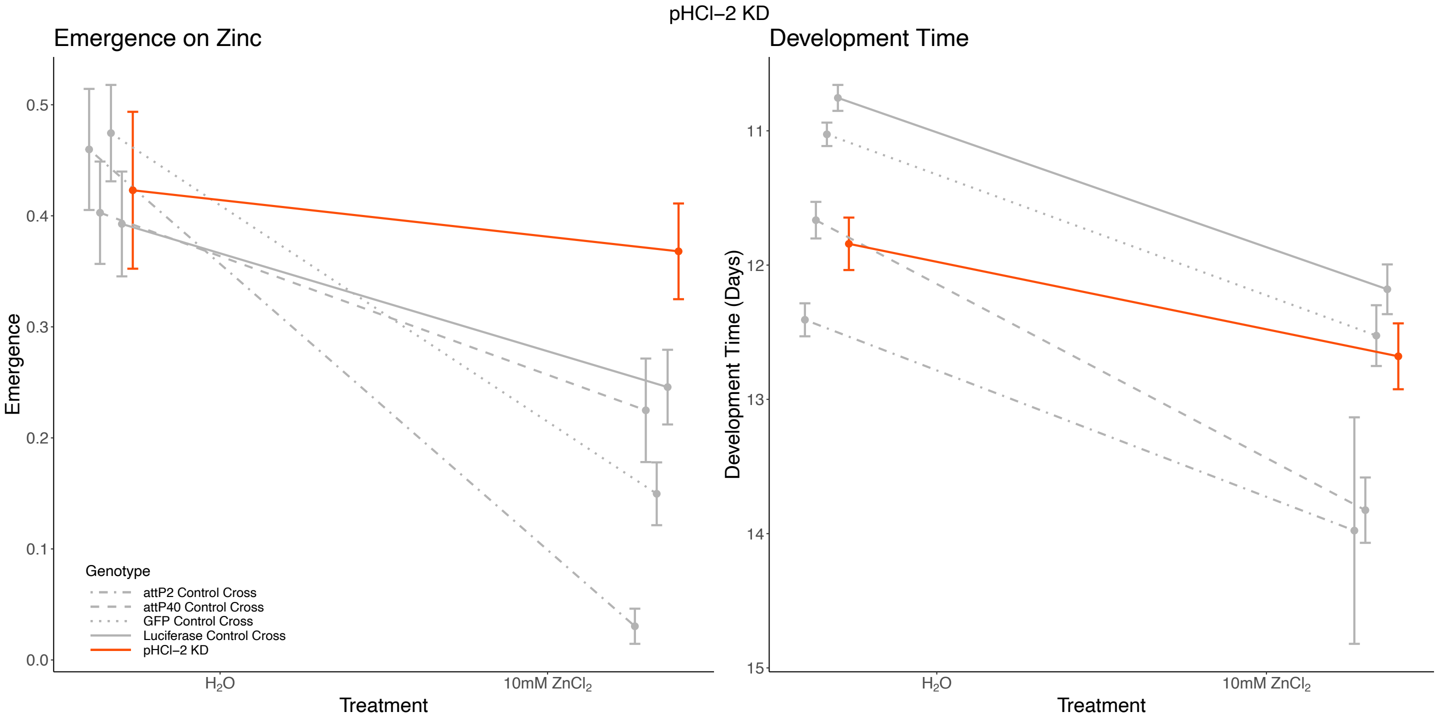

Supplementary Figure 5: Adult zinc toxicity.

Adult females from the X-QTL subpopulations (R9C, R9Z, R10C, R10Z) and from the phenotyping-only cohort were raised in normal lab media, and placed into vials with instant media reconstituted with 100mM $ZnCl_2$. The number of dead flies in a vial was recorded each day untill all flies had died. Below we plot the mean time of death and 95% confidence interval for each sample of flies. Adults from the zinc-selected populations either showed no difference in adult resistance, or were slightly less resistant to zinc than non-selected control populations. (We note that flies from each test population were also put in vials reconstituted with water rather than a zinc chloride solution, and no flies died in these vials over the course of the assay).

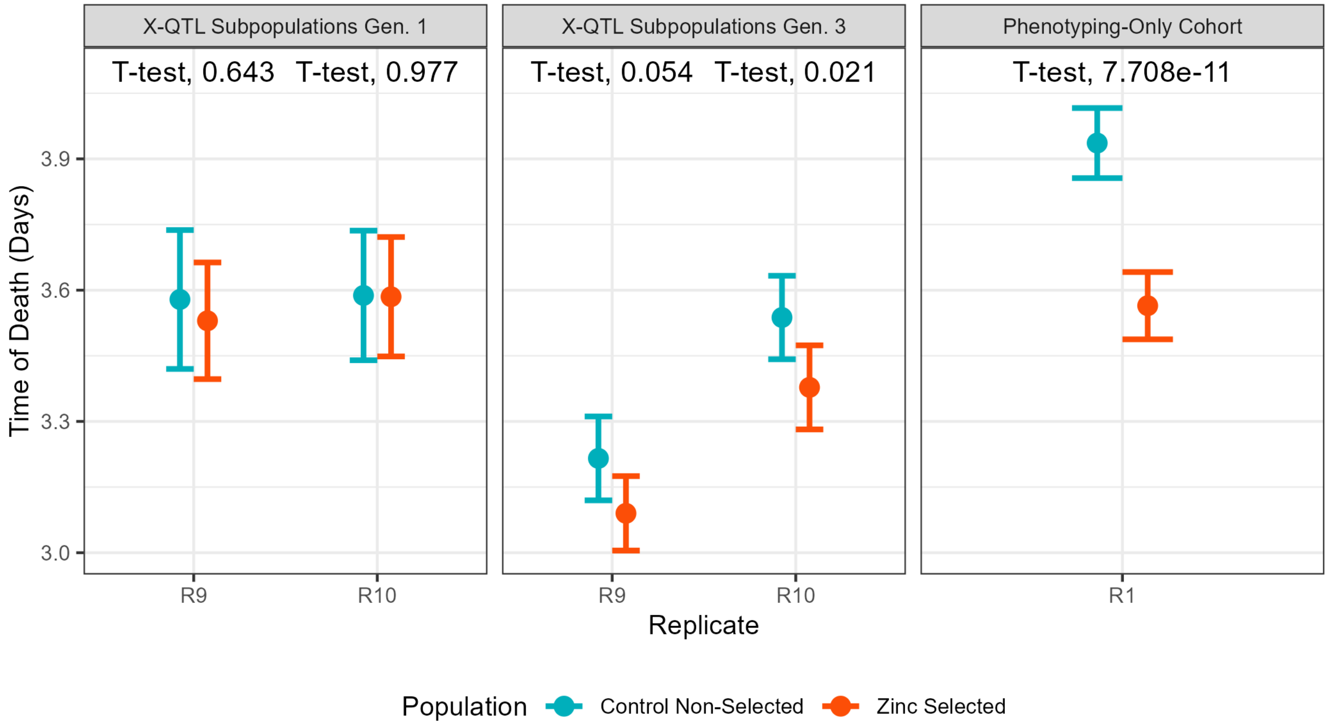

Supplementary Figure 6: Emergence rates of flies from media containing different metal salts.

For the phenotyping-only cohorts and the semi-inbred lines, we collected multiple replicate vials of 50 eggs. Vials contained instant media reconstituted either with water or with a different metal solution (25 or 10mM $ZnCl_2$, 0.2mM $CdCl_2$, or 2mM $CuSO_4$). We counted the number of adults emerging from each vial, and in the panels below points represent vial-specific emergence fractions. (A) Phenotyping-only cohorts. We generated outbred animals in an analogous fashion to our X-QTL design. The zinc-selected cohorts showed higher emergence on zinc media than did non-selected controls ($p = 0.017$), but lower emergence on cadmium media ($p < 1 \times 10^{-5}$), and no difference on copper ($p > 0.05$). (B) Semi-inbred Lines. Strains derived from the R9 and R10 X-QTL control and zinc-selected cohorts showed no significant population-by-treatment interaction for any metal. (The H_2O and $ZnCl_2$ data is identical to that presented in Figure 3B and 3C).

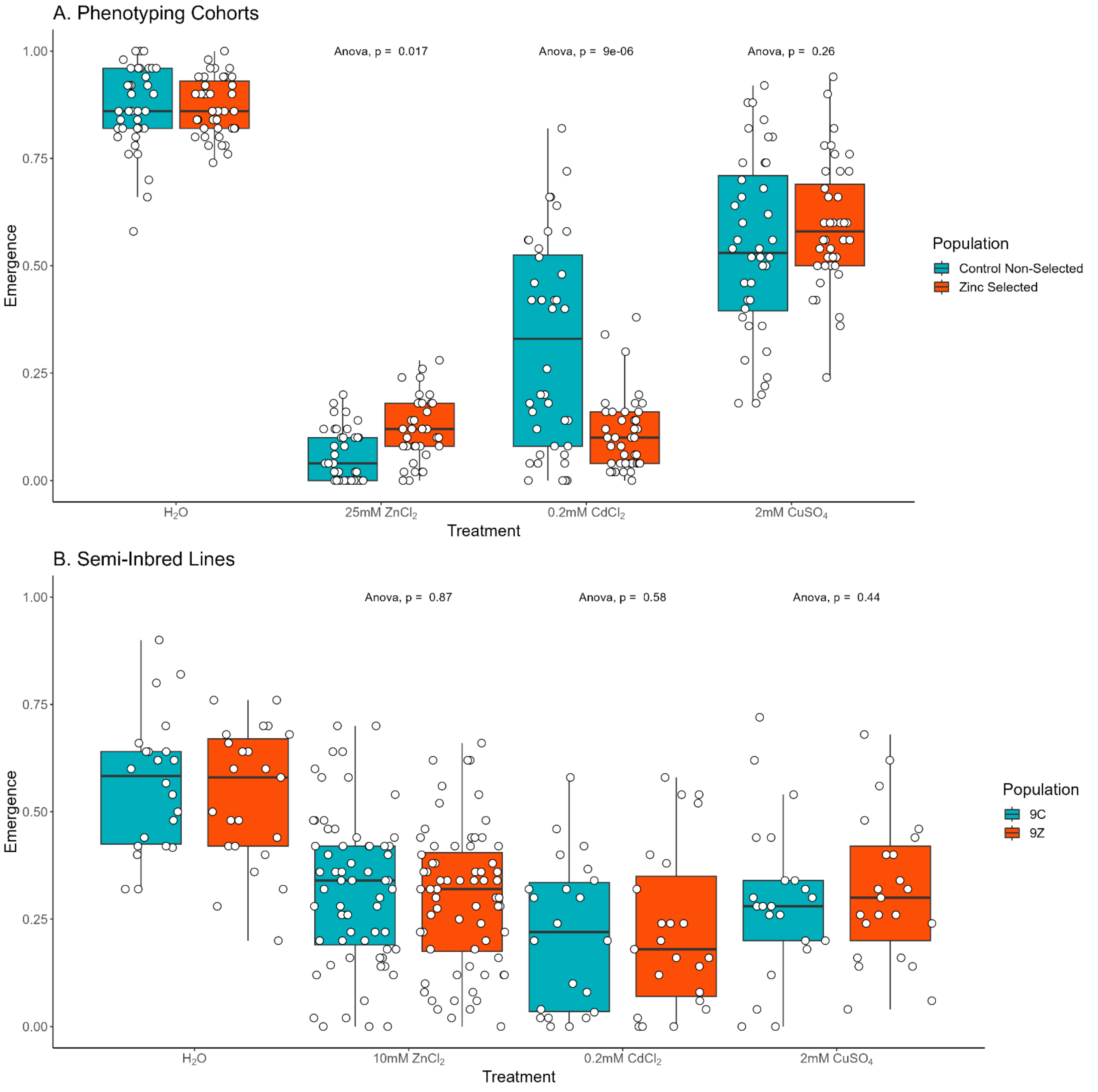

Supplementary Figure 7: Development time of flies in media containing different metal salts.

For the phenotyping-only cohorts and the semi-inbred lines, we collected multiple replicate vials of 50 eggs. Vials contained instant media reconstituted either with water or with a different metal solution (25 or 10mM $ZnCl_2$, 0.2mM $CdCl_2$, or 2mM $CuSO_4$). We measured development time from egg collection to adult emergence, and the points in the figures below represent development time of individual animals. (A) Phenotyping-only cohorts. We generated outbred animals in an analogous fashion to our X-QTL design. We see no difference in development between zinc-selected and control animals on zinc media ($p = 0.34$), while zinc-selected animals experience greater developmental delays due to both cadmium ($p < 1 \times 10^{-59}$) and copper ($p < 1 \times 10^{-11}$). (B) Semi-inbred Lines. Strains derived from the R9 and R10 X-QTL control and zinc-selected cohorts showed a significant population-by-treatment interaction effect for zinc, but not for cadmium or copper. (The H_2O and $ZnCl_2$ data is identical to that presented in Figure 4B and 4C).

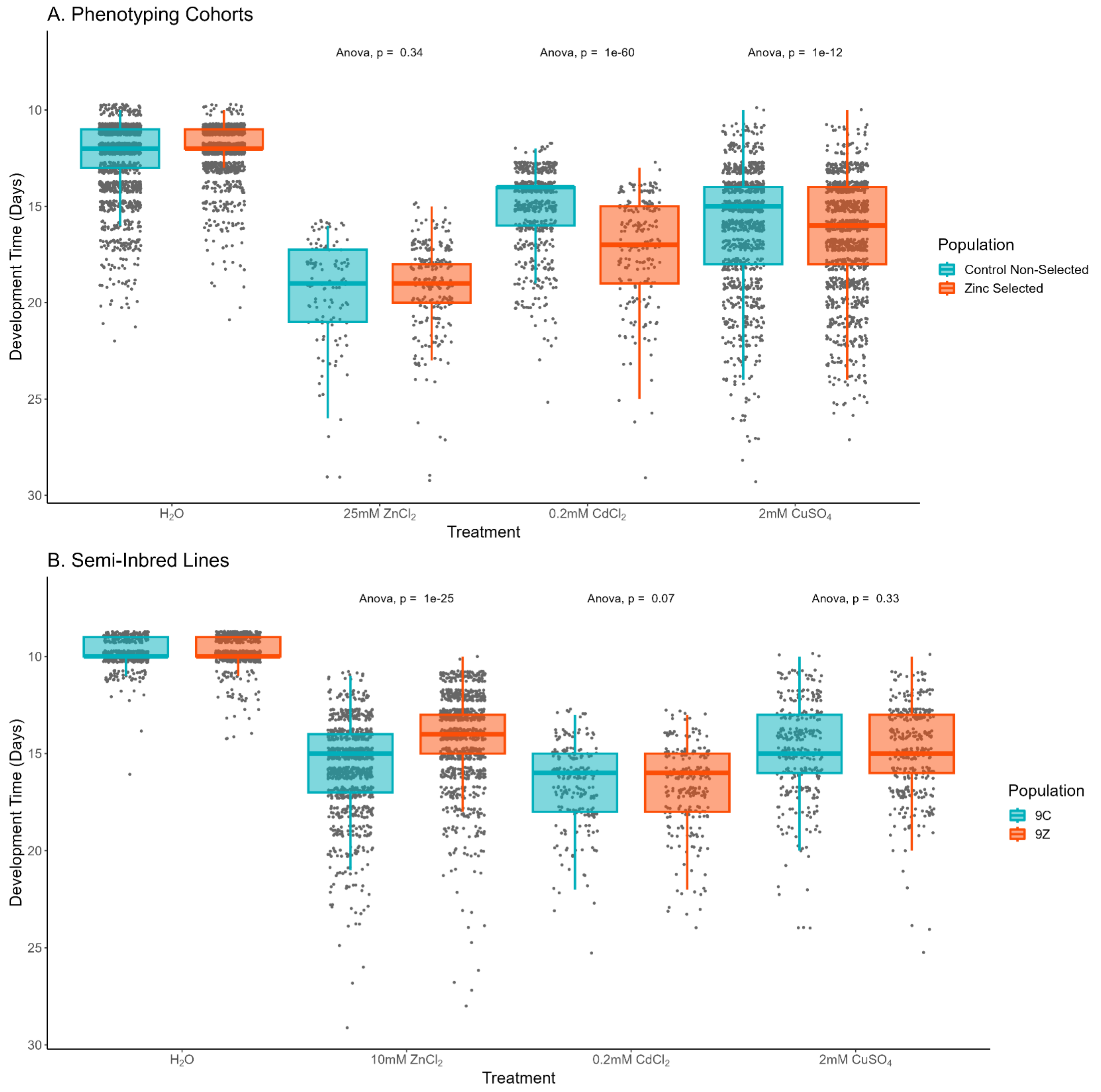

Supplementary Table 9: Set of genes known to be involved in zinc homeostasis, including zinc transporter genes (derived form Navarro & Schneuwly 2017; PMID 29312444). In the "Class" column abbreviations refer to: ZnT = zinc transporter; ZIP = zinc importer; Mtn = methallothionein; TF = transcription factor. In the "Location" column the position of the gene in the Release 6 version of the *D. melanogaster* reference genome is presented.

**Gene**	**Class**	**Chr**	**Location**
ZnT33D	ZnT	chr2L	12164241-12166704
ZnT35C	ZnT	chr2L	15228728-15247714
ZnT41F	ZnT	chr2R	5774776-5782858
ZnT49B	ZnT	chr2R	12652805-12655743
ZnT63C	ZnT	chr3L	3303708-3314009
ZnT77C	ZnT	chr3L	20484293-20494921
ZnT86D	ZnT	chr3R	11212308-11214539
Catsup	ZIP	chr2L	19041862-19043614
foi	ZIP	chr3L	8525244-8534508
Zip42C.1	ZIP	chr2R	6716342-6717707
Zip42C.2	ZIP	chr2R	6718196-6719533
Zip48C	ZIP	chr2R	11899517-11901394
Zip71B	ZIP	chr3L	14992289-14997983
Zip88E	ZIP	chr3R	15268662-15270532
Zip89B	ZIP	chr3R	16305758-16308700
Zip99C	ZIP	chr3R	29863701-29868895
Zip102B	ZIP	chr4	310930-314087
MtnA	Mtn	chr3R	9783407-9784370
MtnB	Mtn	chr3R	20503102-20503746
MtnC	Mtn	chr3R	20360308-20360634
MtnD	Mtn	chr3R	20534962-20535301
MtnE	Mtn	chr3R	20534021-20534704
MtnF	Mtn	chr3R	24634625-24635165
MTF-1	TF	chr3L	9430312-9439490
